# Supplementary material for: The Application of Systems Thinking to the Prevention and Control of Sexually Transmissible Infections among Adolescents and Adults: A Scoping Review
Source: Int J Environ Res Public Health. 2023 May 2;20(9):5708. doi: 10.3390/ijerph20095708 (PMC10178699; doi:10.3390/ijerph20095708)
Supplement: Supplementary file 1 [file ijerph-20-05708-s001.zip › ijerph-2326226-supplementary.pdf]

Table S1: Search strategy and results, by database

Database and date searched	Search strategy	Results
**Medline (Ovid)** 22 December 2022	1. "sexually transmi*".mp.	51535
	2. STD*.mp.	15472
	3. STI.mp.	11355
	4. STIs.mp.	7088
	5. venereal.mp.	7201
	6. HIV.mp.	394132
	7. "human immunodeficiency virus*".mp.	106964
	8. chlamydia.mp.	32063
	9. syphilis.mp.	37325
	10. "acquired immune deficiency syndrome".mp.	6296
	11. "acquired immunodeficiency syndrome".mp.	92289
	12. "sexual health".mp.	13857
	13. "reproductive health".mp.	21891
	14. gonorr*.mp.	26582
	15. exp Sexually Transmitted Diseases/	373347
	16. exp HIV/	106401
	17. exp Chlamydia Infections/ or exp Chlamydia/ or exp Chlamydia trachomatis/	26635
	18. exp Gonorrhea/ or exp Neisseria gonorrhoeae/	19904
	19. exp Syphilis/ or exp Syphilis, Congenital/	29312
	20. exp Acquired Immunodeficiency Syndrome/	78138
	21. exp Sexual Health/	2276
	22. 1 or 2 or 3 or 4 or 5 or 6 or 7 or 8 or 9 or 10 or 11 or 12 or 13 or 14 or 15 or 16 or 17 or 18 or 19 or 20 or 21	589429
	23. "system* thinking".mp.	1563
	24. "system* science".mp.	801
	25. "system* dynamics".mp.	3211
	26. "system* model*".mp.	7655
	27. "system* analys*".mp.	23360
	28. "system* theor*".mp.	7261
	29. "system* approach*".mp.	21101
	30. "system* medicine*".mp.	681
	31. "system* oriented approach".mp.	73
	32. "system*-oriented approach".mp.	73
	33. "complex* science".mp.	425
	34. "complex system*".mp.	12982
	35. "complexity theor*".mp.	528
	36. "adaptive system*".mp.	1717
	37. "system* lens".mp.	110
	38. "system* perspective".mp.	2999
	39. "soft system*".mp.	214

	40. "big-picture thinking".mp.	13
	41. "big picture thinking".mp.	13
	42. "design thinking".mp.	455
	43. "hierarchical thinking".mp.	13
	44. "holistic thinking".mp.	75
	45. "multidimensional thinking".mp.	6
	46. "multi-dimensional thinking".mp.	2
	47. exp Systems Analysis/	101549
	48. exp Systems Theory/	3081
	49. 23 or 24 or 25 or 26 or 27 or 28 or 29 or 30 or 31 or 32 or 33 or 34 or 35 or 36 or 37 or 38 or 39 or 40 or 41 or 42 or 43 or 44 or 45 or 46 or 47 or 48	173064
	50. 22 AND 49	1962
	51. limit 50 to (english language and yr="2010 - Current")	1258
**Cochrane CENTRAL** 22 December 2022	1. MeSH descriptor: [Sexually Transmitted Diseases] explode all trees	15457
	2. (sexually NEXT transmi*):ti,ab,kw	2907
	3. STD:ti,ab,kw	1211
	4. STI:ti,ab,kw	1526
	5. venereal:ti,ab,kw	86
	6. HIV:ti,ab,kw	29531
	7. MeSH descriptor: [HIV] explode all trees	3291
	8. (human NEXT immunodeficiency):ti,ab,kw	13253
	9. chlamydia:ti,ab,kw	1823
	10. MeSH descriptor: [Chlamydia] explode all trees	362
	11. gonorrh*ea:ti,ab,kw	1325
	12. MeSH descriptor: [Gonorrhea] explode all trees	513
	13. syphilis:ti,ab,kw	809
	14. MeSH descriptor: [Syphilis] explode all trees	169
	15. (acquired NEXT immune):ti,ab,kw	1166
	16. MeSH descriptor: [Acquired Immunodeficiency Syndrome] explode all trees	2063
	17. (sexual NEXT health):ti,ab,kw	1559
	18. MeSH descriptor: [Sexual Health] explode all trees	84
	19. (reproductive NEXT health):ti,ab,kw	1662
	20. #1 or #2 or #3 or #4 #5 or #6 or #7 or #8 or #9 or #10 or #11 or #12 or #13 or #14 or #15 #16 or #17 or #18 or #19	37456
	21. (system* NEXT thinking):ti,ab,kw	28
	22. (system* NEXT science):ti,ab,kw	9
	23. (system* NEXT dynamics):ti,ab,kw	27
	24. (system* NEXT model):ti,ab,kw	161
	25. (system* NEXT analysis):ti,ab,kw	385

	26. (system* NEXT theory):ti,ab,kw	128
	27. (system* NEXT approach):ti,ab,kw	552
	28. (system* NEXT medicine):ti,ab,kw	21
	29. (system NEXT oriented):ti,ab,kw	7
	30. (complex* NEXT science):ti,ab,kw	12
	31. (complex NEXT system*):ti,ab,kw	109
	32. (complexity NEXT theor*):ti,ab,kw	7
	33. (adaptive NEXT system*):ti,ab,kw	31
	34. (system* NEXT lens):ti,ab,kw	14
	35. (system* NEXT perspective):ti,ab,kw	395
	36. (soft NEXT system*):ti,ab,kw	6
	37. (big NEXT picture NEXT thinking):ti,ab,kw	0
	38. "design thinking":ti,ab,kw	23
	39. "hierarchical thinking":ti,ab,kw	0
	40. "holistic thinking":ti,ab,kw	8
	41. "multidimensional thinking":ti,ab,kw	0
	42. MeSH descriptor: [Systems Analysis] explode all trees	1906
	43. MeSH descriptor: [Systems Theory] explode all trees	22
	44. #21 #23 or #24 or #25 or #26 or #27 or #28 #29 #31 or #32 or #33 or #34 or #35 or #36 #37 or #38 or #39 or #40 or #41 #42 or #43	1675
	45. #20 AND #44 with Cochrane Library publication date Between Jan 2010 and Dec 2022	68
**EMBASE (Ovid)** 22 December 2022	1. "sexually transmi*".mp.	68658
	2. STD*.mp.	23428
	3. STI.mp.	20767
	4. STIs.mp.	10772
	5. venereal.mp.	6184
	6. HIV.mp.	453911
	7. "human immunodeficiency virus*".mp.	497256
	8. chlamydia.mp.	42215
	9. syphilis.mp.	36919
	10. "acquired immune deficiency syndrome".mp.	142109
	11. "acquired immunodeficiency syndrome".mp.	23493
	12. "sexual health".mp.	28044
	13. "reproductive health".mp.	33328
	14. gonorr*.mp.	33949
	15. exp Sexually Transmitted Diseases/	98968
	16. exp HIV/	215231
	17. exp Chlamydia Infections/ or exp Chlamydia/ or exp Chlamydia trachomatis/	41348
	18. exp Gonorrhea/ or exp Neisseria gonorrhoeae/	31058
	19. exp Syphilis/ or exp Syphilis, Congenital/	30219
	20. exp Acquired Immunodeficiency Syndrome/	152343

	21. exp Sexual Health/	20294
	22. 1 or 2 or 3 or 4 or 5 or 6 or 7 or 8 or 9 or 10 or 11 or 12 or 13 or 14 or 15 or 16 or 17 or 18 or 19 or 20 or 21	790365
	23. "system* thinking".mp.	1766
	24. "system* science".mp.	923
	25. "system* dynamics".mp.	3302
	26. "system* model*".mp.	8872
	27. "system* analys*".mp.	41838
	28. "system* theor*".mp.	7191
	29. "system* approach*".mp.	26910
	30. "system* medicine*".mp.	995
	31. "system* oriented approach".mp.	94
	32. "system*-oriented approach".mp.	94
	33. "complex* science".mp.	473
	34. "complex system*".mp.	13593
	35. "complexity theor*".mp.	567
	36. "adaptive system*".mp.	1881
	37. "system* lens".mp.	122
	38. "system* perspective".mp.	4421
	39. "soft system*".mp.	217
	40. "big-picture thinking".mp.	13
	41. "big picture thinking".mp.	13
	42. "design thinking".mp.	527
	43. "hierarchical thinking".mp.	14
	44. "holistic thinking".mp.	92
	45. "multidimensional thinking".mp.	8
	46. "multi-dimensional thinking".mp.	2
	47. exp Systems Analysis/	21846
	48. exp Systems Theory/	2964
	49. 23 or 24 or 25 or 26 or 27 or 28 or 29 or 30 or 31 or 32 or 33 or 34 or 35 or 36 or 37 or 38 or 39 or 40 or 41 or 42 or 43 or 44 or 45 or 46 or 47 or 48	107289
	50. 22 and 49	1859
	51. limit 50 to (english language and yr="2010 - Current")	1380
**Web of Science** 22 December 2022	1. (((((((((((((((AB=(sexually NEAR/2 transmi*)) OR ALL=(STD*)) OR ALL=(STI)) OR ALL=(STIs)) OR ALL=(venereal)) OR ALL=(HIV)) OR AB=(human NEAR/1 immunodeficiency NEAR/1 virus*)) OR ALL=(chlamydia)) OR ALL=(syphilis)) OR AB=(acquired NEAR/1 immun*)) OR AB=(sexual NEAR/1 health)) OR AB=(reproductive NEAR/1 health)) OR ALL=(gonorr*)) OR TI=("sexually transmi*")) OR TI=("human immun*")) OR TI=("acquired immun*")) OR TI=("sexual health"))	350002

	OR TI=("reproductive health") Timespan: 2010-01-01 to 2022-12-22 Date Run: Thu Dec 22 2022 08:38:03 GMT+0800 (Australian Western Standard Time)	
	2. (((AB=(system* NEAR/1 thinking)) OR AB=(system* NEAR/1 science)) OR AB=(system* NEAR/1 dynamics)) OR AB=(system* NEAR/1 model)) OR AB=(system* NEAR/1 analys*)) OR AB=(system* NEAR/1 theor*)) OR AB=(system* NEAR/1 approach*)) OR AB=(system* NEAR/1 medicine)) OR AB=(system* NEAR/1 oriented)) OR AB=(complex* NEAR/1 science)) OR AB=(complex NEAR/1 system*)) OR AB=(complexity NEAR/1 theor*)) OR AB=(adaptive NEAR/1 system)) OR AB=(system* NEAR/1 lens)) OR AB=(system* NEAR/1 perspective)) OR AB=(soft NEAR/1 system*)) OR AB=(big NEAR/1 picture)) OR AB=(design NEAR/1 thinking)) OR AB=(hierarchical NEAR/1 thinking)) OR AB=(holistic NEAR/1 thinking)) OR AB=(multi*dimensional NEAR/1 thinking)) OR TI=("system* thinking")) OR TI=("system* science")) OR TI=("system* dynamics")) OR TI=("system* model")) OR TI=("system* analysis")) OR TI=("system* theor*")) OR TI=("system* approach*")) OR TI=("system* medicine*")) OR TI=("system* oriented")) OR TI=("complex* science")) OR TI=("complex system*")) OR TI=("complexity theor*")) OR TI=("adaptive system*")) OR TI=("system* lens")) OR TI=("system* perspective")) OR TI=("soft system*")) OR TI=("big picture")) OR TI=("design thinking")) OR TI=("hierarchical thinking")) OR TI=("holistic thinking")) OR TI=("multi*dimensional thinking") Timespan: 2010-01-01 to 2022-12-22 Date Run: Thu Dec 22 2022 08:51:12 GMT+0800 (Australian Western Standard Time)	480265
	3. #1 AND #2	3197
**Google Scholar** 22 December 2022	1. STI HIV Systems Thinking	200

Supplementary Document S1: List of studies excluded at full-text screening

Aantjes CJ, Burrows D, Armstrong R. Capacity development in pursuit of social change: an examination of processes and outcomes. Development in Practice [Internet]. 2022;32(4):536–50.

Reason for exclusion: Ineligible condition

Abbate JL, Murall CL, Richner H, Althaus CL. Potential Impact of Sexual Transmission on Ebola Virus Epidemiology: Sierra Leone as a Case Study. PLoS Neglected Tropical Diseases [electronic resource] [Internet]. 2016;10(5):e0004676.

Reason for exclusion: Ineligible phenomena of interest

Abdullah MA, Shaikh BT, Ghazanfar H. Curing or causing? HIV/AIDS in health care system of Punjab, Pakistan. PLoS ONE [Electronic Resource] [Internet]. 2021;16(7):e0254476.

Reason for exclusion: Ineligible phenomena of interest

Abdullah MA, Shaikh BT. Confusion and denial: need for systems thinking to understand the HIV epidemic in Pakistan. Journal of Ayub Medical College, Abbottabad: JAMC [Internet]. 2014;26(3):396–400.

Reason for exclusion: Ineligible study design

Abdullah MA, Shaikh BT. Review of HIV response in Pakistan using a system thinking framework. Global health action [Internet]. 2015;8:25820.

Reason for exclusion: Ineligible study design

Adam PC, de Wit JB, Bourne CP, Knox D, Purchas J. Promoting regular testing: an examination of HIV and STI testing routines and associated socio-demographic, behavioral and social-cognitive factors among men who have sex with men in New South Wales, Australia. AIDS and behavior. 2014;18(5):921–32.

Reason for exclusion: Ineligible phenomena of interest

Adams JW, Khan MR, Bessey SE, Friedman SR, McMahon JM, Lurie MN, et al. Preexposure prophylaxis strategies for African-American women affected by mass incarceration. AIDS [Internet]. 2021;35(3):453–62.

Reason for exclusion: Ineligible phenomena of interest

Adams OP, Carter AO, Redwood-Campbell L. Understanding attitudes, barriers and challenges in a small island nation to disease and partner notification for HIV and other sexually transmitted infections: a qualitative study. BMC Public Health. 2015;15(1):1–9.

Reason for exclusion: Ineligible phenomena of interest

Adeoti AO, Demir E, Adeyemi S, Yakutcan U, Kengne AP, Kayode G, et al. Impact of pre-exposure and post-exposure prophylaxes prevention programme on HIV burden and services in a low-resource setting: a simulation modelling approach. The Pan African medical journal [Internet]. 2021;40:163.

Reason for exclusion: Ineligible phenomena of interest

Agadjanian V, Markosyan K, Team C-A. Labor migration and STI/HIV risks in Armenia: assessing prevention needs and designing effective interventions. Caucasus Research Resource Centers, Yerevan, Armenia. 2013;

Reason for exclusion: Ineligible phenomena of interest

Agadjanian V, Zotova N. Structure, culture, and HIV/STI vulnerabilities among migrant women in Russia. In: Immigration and Health. 2019.

Reason for exclusion: Ineligible phenomena of interest

Ahmad FA, Dickey V, Tetteh EK, Foraker R, McKay VR. The Use of the Consolidated Framework for Implementation Research to Understand Facilitators and Barriers to Sexually Transmitted Infection Screening in Primary Care. Sexually Transmitted Diseases [Internet]. 2022;49(9):610–5.

Reason for exclusion: Ineligible phenomena of interest

Ahrens KR, Spencer R, Bonnar M, Coatney A, Hall T. Qualitative evaluation of historical and relational factors influencing pregnancy and sexually transmitted infection risks in foster youth. Children and Youth Services Review [Internet]. 2016;61:245–52.

Reason for exclusion: Ineligible phenomena of interest

Alfaro-Ponce M, Arguelles A, Chairez I. Continuous neural identifier for uncertain nonlinear systems with time delays in the input signal. Neural Networks [Internet]. 2014;60:53–66.

Reason for exclusion: Ineligible context

Aliabadi N, Carballo-Dieguez A, Bakken S, Rojas M, Brown III W, Carry M, et al. Using the information-motivation-behavioral skills model to guide the development of an HIV prevention smartphone application for high-risk MSM. AIDS education and prevention: official publication of the International Society for AIDS Education. 2015;27(6):522.

Reason for exclusion: Ineligible phenomena of interest

Allen S, Kilembe W, Inambao M, Wall K, Vwalika B, Appiagey A, et al. From research to policy to implementation: A national plan for couples' voluntary HIV counseling and testing (CVCT) in Zambia. AIDS Research and Human Retroviruses [Internet]. 2016;32(Supplement 1):74.

Reason for exclusion: Ineligible study design

Althaus CL, De Boer RJ. Intracellular transactivation of HIV can account for the decelerating decay of virus load during drug therapy. Molecular Systems Biology [Internet]. 2010;6:348.

Reason for exclusion: Ineligible context

Aminullah E. STI policy and R&D governance for the attainment of SDGs: envisioning the Indonesia's future. Asian Journal of Technology Innovation [Internet]. 2020;28(2):204–33.

Reason for exclusion: Ineligible condition

Amiri S, Pham CD, Amram O, Alcover KC, Oluwoye O, Bravo L, et al. Proximity to Screening Site, Rurality, and Neighborhood Disadvantage: Treatment Status among Individuals with Sexually Transmitted Infections in Yakima County, Washington. International Journal of Environmental Research and Public Health [Internet]. 2020;17(8).

Reason for exclusion: Ineligible phenomena of interest

Anderson J. Workforce considerations in getting to zero. Sexually Transmitted Infections [Internet]. 2021;97(SUPPL 1):A1.

Reason for exclusion: Ineligible study design

Anderson S, Shannon K, Li J, Lee Y, Chettiar J, Goldenberg S, et al. Condoms and sexual health education as evidence: impact of criminalization of in-call venues and managers on migrant sex workers access to HIV/STI prevention in a Canadian setting. BMC international health and human rights. 2016;16(1):1–10.

Reason for exclusion: Ineligible phenomena of interest

Andrews MM, Storm DS, Burr CK, Aaron E, Hoyt MJ, Statton A, et al. Perinatal HIV Service Coordination: Closing Gaps in the HIV Care Continuum for Pregnant Women and Eliminating Perinatal HIV Transmission in the United States. Public Health Reports [Internet]. 2018;133(5):532–42.

Reason for exclusion: Ineligible phenomena of interest

Anku PJ, Amo-Adjei J, Doku D, Kumi-Kyereme A. Challenges of scaling-up of TB-HIV integrated service delivery in Ghana. PLoS ONE [Electronic Resource] [Internet]. 2020;15(7):e0235843.

Reason for exclusion: Ineligible phenomena of interest

Annadurai K, Bagavandas M. A systematic approach for early identification of Syphilis Infection among Men having Sex with Men in Chennai City: - A Page Rank Based Approach. Indian Journal of Community Health. 2017;29(4):389–95.

Reason for exclusion: Duplicate study

Apenteng OO, Ismail NA. A Markov Chain Monte Carlo Approach to Estimate AIDS after HIV Infection. PLoS ONE [Electronic Resource] [Internet]. 2015;10(7):e0131950.

Reason for exclusion: Ineligible phenomena of interest

Apidechkul T. An effectiveness of HIV/AIDS prevention model in akha youths, Thailand. Journal of Microbiology, Immunology and Infection [Internet]. 2015;1):S34.

Reason for exclusion: Ineligible study design

Aral SO, Leichliter JS, Blanchard JF. Overview: the role of emergent properties of complex systems in the epidemiology and prevention of sexually transmitted infections including HIV infection. Sexually Transmitted Infections [Internet]. 2010;86 Suppl 3:iii1-3.

Reason for exclusion: Ineligible study design

Armenta RF, Kellogg D, Montoya JL, Romero R, Armao S, Calac D, et al. â◎◎There is a lot of

practice in not thinking about thatâ◎◎: Structural, interpersonal, and individual-level barriers to HIV/STI prevention among Reservation based American Indians. International Journal of Environmental Research and Public Health. 2021;18(7):3566.

Reason for exclusion: Ineligible phenomena of interest

Armitage G, Hodgson I, Wright J, Bailey K, Mkhwana E. Exploring the delivery of antiretroviral therapy for symptomatic HIV in Swaziland: threats to the successful treatment and safety of outpatients attending regional and district clinics. BMJ Quality & Safety [Internet]. 2011;20(1):52–9.

Reason for exclusion: Ineligible phenomena of interest

Arnold EA, Hazelton P, Lane T, Christopoulos KA, Galindo GR, Steward WT, et al. A qualitative study of provider thoughts on implementing pre-exposure prophylaxis (PrEP) in clinical settings to prevent HIV infection. PLoS ONE. 2012;7(7):e40603.

Reason for exclusion: Ineligible phenomena of interest

Arrington-Sanders R, Ellen J. A systems approach to improve human immunodeficiency virus screening in sexually active youth in urban academic ambulatory settings. Sexually Transmitted Diseases [Internet]. 2011;38(8):777–9.

Reason for exclusion: ineligible publication date

Atinga RA, Agyepong IA, Esena RK. Willing but unable? Extending theory to investigate community capacity to participate in Ghana's community-based health planning and service implementation. Evaluation and program planning [Internet]. 2019;72:170–8.

Reason for exclusion: Ineligible condition

Auerbach JD, Forsyth AD, Davey C, Hargreaves JR, Group for lessons from pandemic HIV prevention for the C. Living with COVID-19 and preparing for future pandemics: revisiting lessons from the HIV pandemic. The Lancet HIV [Internet]. 2022;9:9.

Reason for exclusion: Ineligible study design

Bailey SL, Bono RS, Nash D, Kimmel AD. Implementing parallel spreadsheet models for health policy decisions: The impact of unintentional errors on model projections. PLoS ONE [Electronic Resource] [Internet]. 2018;13(3):e0194916.

Reason for exclusion: Ineligible phenomena of interest

Bain LE, Tarkang EE, Ebuenyi ID, Kamadjeu R. The HIV/AIDS pandemic will not end by the year 2030 in low and middle income countries. The Pan African medical journal [Internet]. 2019;32:67.

Reason for exclusion: Ineligible study design

Baker C, Limato R, Tumbelaka P, Rewari BB, Nasir S, Ahmed R, et al. Antenatal testing for anaemia, HIV and syphilis in Indonesia - a health systems analysis of low coverage. BMC Pregnancy & Childbirth [Internet]. 2020;20(1):326.

Reason for exclusion: Ineligible phenomena of interest

Baraitser P, Holdsworth G, Joia DS, Free C. Contemporary technology for patientcentred innovation - A mixed method evaluation in sexual health. International journal for quality in health care [Internet]. 2017;29:54â◎◎.

Reason for exclusion: Ineligible phenomena of interest

Barbee LA, Khosropour CM, Dombrowksi JC, Golden MR. New HIV diagnosis independently associated with rectal gonorrhea and chlamydia in men who have sex with men. Sexually Transmitted Diseases. 2017;44(7):385.

Reason for exclusion: Ineligible phenomena of interest

Bautista-Arredondo S, Nance N, Salas-Ortiz A, Akeju D, Oluwayinka AG, Ezirim I, et al. The role of management on costs and efficiency in HIV prevention interventions for female sex workers in Nigeria: A cluster-randomized control trial NCT03371914 NCT. Cost effectiveness and resource allocation [Internet]. 2018;16(1) (no pagination).

Reason for exclusion: Duplicate study

Bautista-Arredondo S, Nance N, Salas-Ortiz A, Akeju D, Oluwayinka AG, Ezirim I, et al. The role of management on costs and efficiency in HIV prevention interventions for female sex workers in Nigeria: a cluster-randomized control trial NCT03371914 NCT 11 Medical and Health Sciences 1117 Public Health and Health Services. Cost effectiveness and resource allocation [Internet]. 2018;16(1).

Reason for exclusion: Duplicate

Bautista-Arredondo S, Nance N, Salas-Ortiz A, Akeju D, Oluwayinka AG, Ezirim I, et al. The role of management on costs and efficiency in HIV prevention interventions for female sex workers in Nigeria: a cluster-randomized control trial. Cost Effectiveness & Resource Allocation [Internet]. 2018;16:37.

Reason for exclusion: Duplicate study

Bavinton BR, Grulich AE. HIV pre-exposure prophylaxis: scaling up for impact now and in the future. Lancet Public Health [Internet]. 2021;6(7):E528–33.

Reason for exclusion: Ineligible study design

Bazira D, Lavoie MC, Marima R, Koech E, Claassen C. Academic institutional impact on global HIV pandemic response: A decade of Implementing PEPFAR programs by University of Maryland Baltimore. Journal of Acquired Immune Deficiency Syndromes [Internet]. 2019;81(Supplement 1):46.

Reason for exclusion: Ineligible study design

Bekker LG, Beyrer C, Quinn TC. Behavioral and biomedical combination strategies for HIV prevention. Cold Spring Harbor Perspectives in Medicine [Internet]. 2012;2(8):1.

Reason for exclusion: Ineligible study design

Bekker LG, Ratevosian J, Spencer J, Piot P, Beyrer C. Governance for health: the HIV response and general global health. Bulletin of the World Health Organization [Internet]. 2019;97(3):170–170A.

Reason for exclusion: Ineligible study design

Beltran LF, Davis DA, Barrington C, Castro M, Perez D. "I did not get this disease on the street; it was brought home as a gift": Gender, violence and HIV vulnerability among Cuban women. Culture, Health & Sexuality [Internet]. 2022;24(8):1107–21.

Reason for exclusion: Ineligible phenomena of interest

Bemelmans M, Baert S, Goemaere E, Wilkinson L, Vandendyck M, van Cutsem G, et al. Community-supported models of care for people on HIV treatment in sub-Saharan Africa. Tropical Medicine & International Health [Internet]. 2014;19(8):968–77.

Reason for exclusion: Ineligible study design

Bernstein KT. Systems Approaches to Improving Rates of Extragenital Chlamydia and Gonorrhea Screening Among Men Who Have Sex With Men Engaged in Human Immunodeficiency Virus Care. Sexually Transmitted Diseases [Internet]. 2015;42(10):599–600.

Reason for exclusion: Ineligible study design

Beyrer C, Sullivan P, Sanchez J, Baral SD, Collins C, Wirtz AL, et al. The increase in global HIV epidemics in MSM. AIDS. 2013;27(17):2665–78.

Reason for exclusion: Ineligible study design

Beyrer C, Sullivan PS, Sanchez J, Dowdy D, Altman D, Trapence G, et al. A call to action for comprehensive HIV services for men who have sex with men. The Lancet. 2012;380(9839):424–38.

Reason for exclusion: Ineligible study design

Bhunu CP, Mushayabasa S. Assessing the effects of drug misuse on HIV/AIDS prevalence. Theory in Biosciences [Internet]. 2013;132(2):83–92.

Reason for exclusion: Ineligible phenomena of interest

Bibi Z, Ahmad J, Ali A, Siddiqa A, Shahzad S, Tareen SHK, et al. On the modeling and analysis of the biological regulatory network of NF-kB activation in HIV-1 infection. Complex Adaptive Systems Modeling [Internet]. 2016;4.

Reason for exclusion: Ineligible context

Bilodeau A, Beauchemin J, Bourque D, Galarneau M. The analysis of complex interventions in public health: the STD prevention case at Montreal. Canadian Journal of Public Health-Revue Canadienne De Sante Publique [Internet]. 2013;104(2):E177–82.

Reason for exclusion: Not English

Birnbaum JM, Loundsbury DW, Eastwood E, Palma A, Jo GY. USE OF SYSTEM DYNAMICS MODELING AS A TOOL FOR EVALUATION OF INTERVENTION PLANNING FOR HIV plus ADOLESCENTS AND THEIR RETENTION IN CARE IN AN URBAN ADOLESCENT HIV CLINIC. Journal of Adolescent Health [Internet]. 2013;52(2):S82–3.

Reason for exclusion: Ineligible study design

Birnbaum JM, Loundsbury DW, Eastwood E, Palma A, Jo GY. USE OF SYSTEM DYNAMICS MODELING AS A TOOL FOR EVALUATION OF INTERVENTION PLANNING FOR HIV plus ADOLESCENTS AND THEIR RETENTION IN CARE IN AN URBAN ADOLESCENT HIV CLINIC. Journal of Adolescent Health [Internet]. 2013;52(2):S82–3.

Reason for exclusion: Duplicate

Birnbaum JM, Loundsbury DW, Eastwood E, Palma A, Jo GY. Use of system dynamics modeling as a tool for evaluation of intervention planning for HIV< adolescents and their retention in care in an Urban adolescent HIV clinic. Journal of Adolescent Health [Internet]. 2013;1):S82–3.

Reason for exclusion: Ineligible study design

Birnbaum JM, Loundsbury DW, Eastwood E, Palma A, Jo GY. Use of system dynamics modeling as a tool for evaluation of intervention planning for HIV< adolescents and their retention in care in an Urban adolescent HIV clinic. Journal of Adolescent Health [Internet]. 2013;1):S82–3.

Reason for exclusion: Duplicate

Black DS, Sun P, Rohrbach LA, Sussman S. Decision-making style and gender moderation of the self-efficacyâ◎◎condom use link among adolescents and young adults: Informing targeted STI/HIV prevention programs. Archives of Pediatrics & Adolescent Medicine. 2011;165(4):320–5.

Reason for exclusion: Ineligible phenomena of interest

Blackstock OJ, Frew P, Bota D, Vo-Green L, Parker K, Franks J, et al. Perceptions of community HIV/STI risk among US women living in areas with high poverty and HIV prevalence rates. Journal of health care for the poor and underserved. 2015;26(3):811.

Reason for exclusion: Ineligible phenomena of interest

Blanchard JF, Aral SO. Emergent properties and structural patterns in sexually transmitted infection and HIV research. Sexually Transmitted Infections [Internet]. 2010;86 Suppl 3:iii4-9.

Reason for exclusion: ineligible publication date

Borawski EA, Tufts KA, Trapl ES, Hayman LL, Yoder LD, Lovegreen LD. Effectiveness of health education teachers and school nurses teaching sexually transmitted infections/human immunodeficiency virus prevention knowledge and skills in high school. Journal of School Health. 2015;85(3):189–96.

Reason for exclusion: Ineligible phenomena of interest

Bouchnita A, Bocharov G, Meyerhans A, Volpert V. Towards a Multiscale Model of Acute HIV Infection. Computation [Internet]. 2017;5(1).

Reason for exclusion: Ineligible context

Boyd MA, Nwizu CA. Operational research in HIV priority areas: the African way. The Lancet [Internet]. 2010;376(9734):4–6.

Reason for exclusion: Ineligible study design

Bradley E, Thompson JW, Byam P, Webster TR, Zerihun A, Alpern R, et al. Access and quality of rural healthcare: Ethiopian Millennium Rural Initiative. International journal for quality in health care [Internet]. 2011;23(3):222–30.

Reason for exclusion: ineligible publication date

Brady M, Manning J. Lessons from reproductive health to inform multipurpose prevention technologies: Don't reinvent the wheel. Antiviral Research [Internet]. 2013;100(SUPPL.):S25–31.

Reason for exclusion: Ineligible study design

Branch M, Harvey SM, Zukoski AP, Warren J. Prevention of unintended pregnancy and HIV/STIs among Latinos in rural communities: Perspectives of health care providers. Health care for women international. 2010;31(8):718–36.

Reason for exclusion: Ineligible phenomena of interest

Broaddus MR, Owczarzak J, Schumann C, Koester KA. Fostering a "Feeling of Worth" Among Vulnerable HIV Populations: The Role of Linkage to Care Specialists. AIDS Patient Care & Stds [Internet]. 2017;31(10):438–46.

Reason for exclusion: Ineligible phenomena of interest

Brotman JS, Mensah FM, Lesko N. Urban high school students' learning about HIV/AIDS in different contexts. Science Education. 2011;95(1):87–120.

Reason for exclusion: Ineligible phenomena of interest

Brown CH, Mohr DC, Gallo CG, Mader C, Palinkas L, Wingood G, et al. A computational future for preventing HIV in minority communities: how advanced technology can improve implementation of effective programs. Journal of Acquired Immune Deficiency Syndromes: JAIDS [Internet]. 2013;63 Suppl 1:S72-84.

Reason for exclusion: Ineligible phenomena of interest

Brown LK, Nugent NR, Houck CD, Lescano CM, Whiteley LB, Barker D, et al. Safe Thinking and Affect Regulation (STAR): Human immunodeficiency virus prevention in alternative/therapeutic schools. Journal of the American Academy of Child & Adolescent Psychiatry. 2011;50(10):1065–74.

Reason for exclusion: Ineligible phenomena of interest

Brown R, Kismodi E, Khosla R, Malla S, Asuagbor L, Andion-Ibanez X, et al. A sexual and reproductive health and rights journey: from Cairo to the present. Sexual and Reproductive Health Matters [Internet]. 2019;27(1) (no pagination).

Reason for exclusion: Ineligible study design

Brunne V, Cooperation Competence N. The Competence Network for HIV/AIDS from a health systems perspective: towards an impact assessment. European Journal of Medical Research [Internet]. 2010;15(8):335–335.

Reason for exclusion: Ineligible study design

Buratto A, Cesaretto R, Zamarchi R. Mathematical models for HIV treatment : A schematic review. Journal of Interdisciplinary Mathematics [Internet]. 2020;23(3):707–25.

Reason for exclusion: Ineligible study design

Burman CJ, Aphane M. Complex HIV/AIDS Landscapes: Reflections on How "Path Creation" Influenced an Action-Oriented Intervention. Systemic Practice and Action Research [Internet]. 2017;30(1):45–66.

Reason for exclusion: Ineligible phenomena of interest

Burman CJ, Aphane MA. Complex adaptive HIV/AIDS risk reduction: Plausible implications from findings in Limpopo Province, South Africa. South African Medical Journal Suid-Afrikaanse Tydskrif Vir Geneeskunde [Internet]. 2016;106(6):48–51.

Reason for exclusion: Ineligible phenomena of interest

Cahill H. DRAMA AS TRANSDISCIPLINARY PRACTICE: USING SYSTEMS THINKING TOOLS TO GENERATE NEW STORIES ABOUT HIV. Nj-Drama Australia Journal [Internet]. 2011;35(1):15–33.

Reason for exclusion: ineligible publication date

Camacho-Gonzalez AF, Wallins A, Toledo L, Murray A, Gaul Z, Sutton MY, et al. Risk factors for HIV transmission and barriers to HIV disclosure: metropolitan Atlanta youth perspectives. AIDS Patient Care and STDs. 2016;30(1):18–24.

Reason for exclusion: Ineligible phenomena of interest

Carter JW, Hart-Cooper GD, Butler MO, Workowski KA, Hoover KW. Provider barriers prevent recommended sexually transmitted disease screening of HIV-infected men who have sex with men. Sexually Transmitted Diseases. 2014;41(2):137–42.

Reason for exclusion: Ineligible phenomena of interest

Cassels S, Goodreau SM. Interaction of mathematical modeling and social and behavioral HIV/AIDS research. Current Opinion in HIV and AIDS [Internet]. 2011;6(2):119–23.

Reason for exclusion: Ineligible study design

Cassidy C, Steenbeek A, Langille D, Martin-Misener R, Curran J. Designing an intervention to improve sexual health service use among university undergraduate students: a mixed methods study guided by the behaviour change wheel. BMC Public Health [Internet]. 2019;19(1):1734.

Reason for exclusion: Ineligible phenomena of interest

Caton CL, El-Bassel N, Gelman A, Barrow S, Herman D, Hsu E, et al. Rates and correlates of HIV and STI infection among homeless women. AIDS and behavior. 2013;17(3):856–64.

Reason for exclusion: Ineligible phenomena of interest

Celletti F, Wright A, Palen J, Frehywot S, Markus A, Greenberg A, et al. Can the deployment

of community health workers for the delivery of HIV services represent an effective and sustainable response to health workforce shortages? Results of a multicountry study. AIDS [Internet]. 2010;24(SUPPL. 1):S45–57.

Reason for exclusion: Ineligible phenomena of interest

Champenois K, Cousien A, Cuzin L, Le Vu S, Deuffic-Burban S, Lanoy E, et al. Missed opportunities for HIV testing in newly-HIV-diagnosed patients, a cross sectional study. BMC Infectious Diseases. 2013;13(1):1–10.

Reason for exclusion: Ineligible phenomena of interest

Chen M, Guy R. Increasing syphilis rates among men who have sex with men and screening to detect asymptomatic infection. Sexual Health [Internet]. 2017;14(4):301–3.

Reason for exclusion: Ineligible study design

Chen XG, Chen DC. Cognitive Theories, Paradigm of Quantum Behavior Change, and Cusp Catastrophe Modeling in Social Behavioral Research. Journal of the Society for Social Work and Research [Internet]. 2019;10(1):127–59.

Reason for exclusion: Ineligible phenomena of interest

Chereshnev VA, Verzilin DN, Imova TGM, Chereshneva EV. Socio-economic monitoring of hiv/aids threat in the Russian Federation. Ekonomika Regiona-Economy of Region [Internet]. 2012;2:153–69.

Reason for exclusion: Not English

Chesang K, Hornston S, Muhenje O, Saliku T, Mirjahangir J, Viitanen A, et al. Healthcare provider perspectives on managing sexually transmitted infections in HIV care settings in Kenya: A qualitative thematic analysis. PLoS Medicine. 2017;14(12):e1002480.

Reason for exclusion: Ineligible phenomena of interest

Claborn K, Jhaveri A, Becker S, Tavares T, Schnarrs P, Nordquist E. CREATING AN HIV/PREP AND SUBSTANCE USE INTEGRATED CARE DIGITAL TOOL USING SERVICE DESIGN THINKING. Annals of Behavioral Medicine [Internet]. 2019;53:S449–S449.

Reason for exclusion: Ineligible study design

Claborn K, Jhaveri A, Becker S, Tavares T, Schnarrs P, Nordquist E. CREATING AN HIV/PREP AND SUBSTANCE USE INTEGRATED CARE DIGITAL TOOL USING SERVICE DESIGN THINKING. Annals of Behavioral Medicine [Internet]. 2019;53:S449–S449.

Reason for exclusion: Duplicate

Clark J, Salvatierra J, Segura E, Salazar X, Konda K, Perez-Brumer A, et al. Moderno love: sexual role-based identities and HIV/STI prevention among men who have sex with men in Lima, Peru. AIDS and behavior. 2013;17(4):1313–28.

Reason for exclusion: Ineligible phenomena of interest

Clark JL, Perez-Brumer A, Salazar X. â◎◎Manejar la Situacionâ◎◎: partner notification, partner management, and conceptual frameworks for HIV/STI control among MSM in Peru.

AIDS and behavior. 2015;19(12):2245–54.

Reason for exclusion: Ineligible phenomena of interest

Cleary S, Erasmus E, Gilson L, Michel C, Gremu A, Sherr K, et al. The everyday practice of supporting health system development: learning from how an externally-led intervention was implemented in Mozambique. Health policy and planning [Internet]. 2018;33(7):801–10.

Reason for exclusion: Ineligible condition

Coetzee B, Kagee A, Vermeulen N. Structural barriers to adherence to antiretroviral therapy in a resource-constrained setting: the perspectives of health care providers. AIDS Care [Internet]. 2011;23(2):146–51.

Reason for exclusion: ineligible publication date

Cohn J, Owiredu MN, Taylor MM, Easterbrook P, Lesi O, Francoise B, et al. Eliminating mother-to-child transmission of human immunodeficiency virus, syphilis and hepatitis B in sub-Saharan Africa. Bulletin of the World Health Organization [Internet]. 2021;99(4):287–95.

Reason for exclusion: Ineligible study design

Coker R, Balen J, Mounier-Jack S, Shigayeva A, Lazarus JV, Rudge JW, et al. A conceptual and analytical approach to comparative analysis of country case studies: HIV and TB control programmes and health systems integration. Health policy and planning [Internet]. 2010;25 Suppl 1:i21-31.

Reason for exclusion: ineligible publication date

Colarossi L, Dean R, Stevens A, Ackeifi J, Noonan M. Sexual and reproductive health capacity building for foster care organizations: A systems model. Children and Youth Services Review [Internet]. 2019;105.

Reason for exclusion: Ineligible phenomena of interest

Cooper D, Mantell JE, Moodley J, Mall S. The HIV epidemic and sexual and reproductive health policy integration: views of South African policymakers. BMC Public Health. 2015;15(1):1–9.

Reason for exclusion: Ineligible phenomena of interest

Cordova D, Alers-Rojas F, Lua FM, Bauermeister J, Nurenberg R, Ovadje L, et al. The usability and acceptability of an adolescent mHealth HIV/STI and drug abuse preventive intervention in primary care. Behavioral medicine. 2018;44(1):36–47.

Reason for exclusion: Ineligible phenomena of interest

Cordova D, Bauermeister JA, Fessler K, Delva J, Nelson A, Nurenberg R, et al. A community-engaged approach to developing an mHealth HIV/STI and drug abuse preventive intervention for primary care: a qualitative study. JMIR MHealth and UHealth. 2015;3(4):e4620.

Reason for exclusion: Ineligible phenomena of interest

Crooks N, Wise A, Frazier T. Addressing sexually transmitted infections in the sociocultural

context of black heterosexual relationships in the United States. Social Science & Medicine. 2020;263:113303.

Reason for exclusion: Ineligible phenomena of interest

Daley AM. Rethinking school-based health centers as complex adaptive systems: maximizing opportunities for the prevention of teen pregnancy and sexually transmitted infections. Advances in Nursing Science [Internet]. 2012;35(2):E37-46.

Reason for exclusion: Ineligible phenomena of interest

Dang MT, Amos A, Dangerfield M, Ford B, Kern K, Moon M. A youth participatory project to address STIs and HIV among homeless youth. Comprehensive child and adolescent nursing. 2019;42(3):222–40.

Reason for exclusion: Ineligible phenomena of interest

Dave G, Ritchwood T, Young TL, Isler MR, Black A, Akers AY, et al. Evaluating teach one reach oneâ◎◎an STI/HIV risk-reduction intervention to enhance adultâ◎◎youth communication about sex and reduce the burden of HIV/STI. American Journal of Health Promotion. 2017;31(6):465–75.

Reason for exclusion: Ineligible phenomena of interest

Davis DA, Aguilar JM, Arandi CG, Northbrook S, Loya-Montiel MI, Morales-Miranda S, et al. "OH, I'M NOT ALONE": EXPERIENCES OF HIV-POSITIVE MEN WHO HAVE SEX WITH MEN IN A HEALTH NAVIGATION PROGRAM TO PROMOTE TIMELY LINKAGE TO CARE IN GUATEMALA CITY. Aids Education and Prevention [Internet]. 2017;29(6):554–66.

Reason for exclusion: Ineligible phenomena of interest

Dawkins CE, Barker JR. A Complexity Theory Framework of Issue Movement. Business & Society [Internet]. 2020;59(6):1110–50.

Reason for exclusion: Ineligible phenomena of interest

De Neve JW, Garrison-Desany H, Andrews KG, Sharara N, Boudreaux C, Gill R, et al. Harmonization of community health worker programs for HIV: A four-country qualitative study in Southern Africa. PLoS Medicine [Internet]. 2017;14(8).

Reason for exclusion: Ineligible phenomena of interest

De Walque D, Dow W, Nathan R. Rewarding safer sex: conditional cash transfers for HIV/STI prevention. World Bank Policy Research Working Paper. 2014;(7099).

Reason for exclusion: Ineligible phenomena of interest

Devaskar S, Stern L, Young A, Leiby S. A DESIGN-THINKING INTERVENTION TO IMPROVE HIV PREVENTION SERVICES IN THE FAMILY PLANNING SETTING. Annals of Behavioral Medicine [Internet]. 2019;53:S59–S59.

Reason for exclusion: Ineligible study design

DiStefano AS. HIV's Syndemic Links With Mental Health, Substance Use, and Violence in an

Environment of Stigma and Disparities in Japan. Qualitative Health Research [Internet]. 2016;26(7):877–94.

Reason for exclusion: Ineligible phenomena of interest

Dilmen E, Beyhan S. Stabilization of HIV Infection Using Deep Recurrent SVM Based Generalized Predictive Control. In: 6th International Conference on Control Engineering and Information Technology (CEIT) [Internet]. 2018.

Reason for exclusion: Ineligible condition

Ding P, Qiu ZP, Li XZ. The population-level impact of HBV and its vaccination on HIV transmission dynamics. Mathematical Methods in the Applied Sciences [Internet]. 2016;39(18):5539–56.

Reason for exclusion: Ineligible phenomena of interest

Doherty T, Besada D, Goga A, Daviaud E, Rohde S, Raphaely N. "If donors woke up tomorrow and said we can't fund you, what would we do?" A health system dynamics analysis of implementation of PMTCT option B plus in Uganda. Globalization and Health [Internet]. 2017;13.

Reason for exclusion: Ineligible phenomena of interest

Doherty T, Besada D, Goga A, Daviaud E, Rohde S, Raphaely N. "If donors woke up tomorrow and said we can't fund you, what would we do?" A health system dynamics analysis of implementation of PMTCT option B+ in Uganda. Global Health [Internet]. 2017;13(1):51.

Reason for exclusion: Duplicate

Doll M, Fortenberry JD, Roseland D, McAuliff K, Wilson CM, Boyer CB. Linking HIV-negative youth to prevention services in 12 US cities: barriers and facilitators to implementing the HIV prevention continuum. Journal of Adolescent Health. 2018;62(4):424–33.

Reason for exclusion: Ineligible phenomena of interest

Doshi RK, Malebranche D, Bowleg L, Sangaramoorthy T. Health care and HIV testing experiences among Black men in the South: implications for â◎◎Seek, Test, Treat, and Retainâ◎◎ HIV prevention strategies. AIDS Patient Care and STDs. 2013;27(2):123–33.

Reason for exclusion: Ineligible phenomena of interest

Draper D. Developing a sector led improvement approach to sexual health. Sexually Transmitted Infections [Internet]. 2016;92(Supplement 1):A30.

Reason for exclusion: Ineligible study design

Eastment M, Wanje G, Richardson B, Mwaringa E, Sherr K, Barnabas R, et al. Results of a cluster randomized trial testing a systems analysis and improvement approach (SAIA) versus usual procedures to increase HIV counseling and testing in family planning clinics in mombasa, Kenya. Implementation science [Internet]. 2021;16(SUPPL 1).

Reason for exclusion: Duplicate study

Eastment MC, Wanje G, Richardson BA, Mwaringa E, Sherr K, Barnabas RV, et al. Results of a cluster randomized trial testing the systems analysis and improvement approach to increase HIV testing in family planning clinics. AIDS (London, England) [Internet]. 2022;36(2):225â©©235.

Reason for exclusion: Duplicate study

Eaton LA, Earnshaw VA, Maksut JL, Thorson KR, Watson RJ, Bauermeister JA. Experiences of stigma and health care engagement among Black MSM newly diagnosed with HIV/STI. Journal of behavioral medicine. 2018;41(4):458–66.

Reason for exclusion: Ineligible phenomena of interest

Edwards N, Barker PM. The importance of context in implementation research. Journal of Acquired Immune Deficiency Syndromes: JAIDS [Internet]. 2014;67 Suppl 2:S157-62.

Reason for exclusion: Ineligible study design

Edwards N, Barker PM. The importance of context in implementation research. Journal of Acquired Immune Deficiency Syndromes: JAIDS [Internet]. 2014;67 Suppl 2:S157-62.

Reason for exclusion: Duplicate study

Elkington KS, Belmonte K, Latack JA, Mellins CA, Wasserman GA, Donenberg GR, et al. An exploration of family and juvenile justice systems to reduce youth HIV/STI risk. Journal of research on adolescence. 2015;25(4):700–16.

Reason for exclusion: Ineligible phenomena of interest

Emmanuel F, Blanchard J, Zaheer HA, Reza T, Holte-McKenzie M, Hasp team. The HIV/AIDS Surveillance Project mapping approach: an innovative approach for mapping and size estimation for groups at a higher risk of HIV in Pakistan. AIDS [Internet]. 2010;24 Suppl 2:S77-84.

Reason for exclusion: Ineligible phenomena of interest

Everhart AR, Boska H, Sinai-Glazer H, Wilson-Yang JQ, Burke NB, LeBlanc G, et al. “I”m not interested in research; i’m interested in services’: How to better health and social services for transgender women living with and affected by HIV. Social Science & Medicine [Internet]. 2022;292.

Reason for exclusion: Ineligible phenomena of interest

Eyre SL, Flythe M, Hoffman V, Fraser AE. Concepts of infidelity among African American emerging adults: Implications for HIV/STI prevention. Journal of Adolescent Research. 2012;27(2):231–55.

Reason for exclusion: Ineligible phenomena of interest

Farahani FK, Akhondi MM, Shirzad M, Azin A. HIV/STI risk-taking sexual Behaviours and risk perception among Male University students in Tehran: implications for HIV prevention among youth. Journal of biosocial science. 2018;50(1):86–101.

Reason for exclusion: Ineligible phenomena of interest

Farel CE, Parker SD, Muessig KE, Grodensky CA, Jones C, Golin CE, et al. Sexuality, sexual practices, and HIV risk among incarcerated African-American women in North Carolina. Women's Health Issues. 2013;23(6):e357–64.

Reason for exclusion: Ineligible phenomena of interest

Faria MJ, Machado R, Ribeiro A, Goncalves H, Oliveira M, Viseu T, et al. Rational Development of Liposomal Hydrogels: A Strategy for Topical Vaginal Antiretroviral Drug Delivery in the Context of HIV Prevention. Pharmaceutics [Internet]. 2019;11(9).

Reason for exclusion: Ineligible context

Farris C, Akers AY, Downs JS, Forbes EE. Translational Research Applications for the Study of Adolescent Sexual Decision Making. Cts-Clinical and Translational Science [Internet]. 2013;6(1):78–81.

Reason for exclusion: Ineligible condition

Fingleton NA, Watson MC, Matheson C. "You are still a human being, you still have needs, you still have wants": a qualitative exploration of patients ' experiences and views of HIV support. Journal of Public Health [Internet]. 2018;40(4):E571–7.

Reason for exclusion: Ineligible phenomena of interest

Fisher CB, Fried AL, Macapagal K, Mustanski B. Patientâ@@provider communication barriers and facilitators to HIV and STI preventive services for adolescent MSM. AIDS and behavior. 2018;22(10):3417–28.

Reason for exclusion: Ineligible phenomena of interest

Flowers P, Vojt G, Pothoulaki M, Mapp F, Owusu MW, Cassell JA, et al. Using the behaviour change wheel approach to optimize self-sampling packs for sexually transmitted infection and blood borne viruses. British Journal of Health Psychology [Internet]. 2022;27(4):1382–97.

Reason for exclusion: Ineligible phenomena of interest

Fogel CI, Gelaude DJ, Carry M, Herbst JH, Parker S, Scheyette A, et al. Context of risk for HIV and sexually transmitted infections among incarcerated women in the south: individual, interpersonal, and societal factors. Women & health. 2014;54(8):694–711.

Reason for exclusion: Ineligible phenomena of interest

Forsyth AD, Yakovchenko V, Valdiserri RO. Opportunities and challenges for an integrated, federal HIV services information system: implications for enhancing HIV programs. Journal of Public Health Management & Practice [Internet]. 2014;20(4):442–4.

Reason for exclusion: Ineligible study design

Frank L, Starzyk E, Hoxworth T, Canon M, McGuinness C, Watkins A, et al. HIV PrEP implementation: A multi-level systems approach. Evaluation and program planning [Internet]. 2022;90:101966.

Reason for exclusion: Ineligible phenomena of interest

Frasca K, Castillo-Mancilla J, McNulty MC, Connors S, Sweitzer E, Zimmer S, et al. A mixed methods evaluation of an inclusive sexual history taking and HIV prevention curriculum for trainees. Journal of General Internal Medicine. 2019;34(7):1279–88.

Reason for exclusion: Ineligible phenomena of interest

Frasca T, Balan I, Ibitoye M, Valladares J, Dolezal C, Carballo-DiÃ©guez A. Attitude and behavior changes among gay and bisexual men after use of rapid home HIV tests to screen sexual partners. AIDS and behavior. 2014;18(5):950–7.

Reason for exclusion: Ineligible phenomena of interest

Freedman LP, Schaaf M. Act global, but think local: accountability at the frontlines. Reproductive Health Matters [Internet]. 2013;21(42):103–12.

Reason for exclusion: Ineligible study design

Freedman LP, Schaaf M. Ad global, but think local: accountability at the frontlines. Reproductive Health Matters [Internet]. 2013;21(42):103–12.

Reason for exclusion: Duplicate

Freeman R, Gwadz MV, Silverman E, Kutnick A, Leonard NR, Ritchie AS, et al. Critical race theory as a tool for understanding poor engagement along the HIV care continuum among African American/Black and Hispanic persons living with HIV in the United States: a qualitative exploration. International Journal for Equity in Health [Internet]. 2017;16.

Reason for exclusion: Ineligible phenomena of interest

Frew PM, Parker K, Vo L, Haley D, Oâ◎◎Leary A, Diallo DD, et al. Socioecological factors influencing womenâ◎◎s HIV risk in the United States: qualitative findings from the womenâ◎◎s HIV SeroIncidence study (HPTN 064). BMC Public Health. 2016;16(1):1–18.

Reason for exclusion: Ineligible phenomena of interest

Friend DR. An update on multipurpose prevention technologies for the prevention of HIV transmission and pregnancy. Expert opinion on drug delivery. 2016;13(4):533–45.

Reason for exclusion: Ineligible study design

Furuoka F, Hoque MZ. Determinants of antiretroviral therapy coverage in Sub-Saharan Africa. PeerJ [Internet]. 2015;3.

Reason for exclusion: Ineligible phenomena of interest

Gaines M, Wiggins J, Sierzant B, Coleman B, Hannah W. Removing barriers to HIV screening in a community-based pediatric emergency department. Sexually Transmitted Infections [Internet]. 2021;97(SUPPL 1):A89.

Reason for exclusion: Ineligible study design

Geetha V, Balamuralitharan S. Stability analysis of host dynamics for hiv. In: 10th National Conference on Mathematical Techniques and its Applications (NCMTA) [Internet]. 2018.

Reason for exclusion: Ineligible context

Gerbi GB, Habtemariam T, Tameru B, Nganwa D, Robnett V. A quantitative risk assessment of multiple factors influencing HIV/AIDS transmission through unprotected sex among HIV-seropositive men. AIDS Care [Internet]. 2012;24(3):331–9.

Reason for exclusion: Ineligible phenomena of interest

Gibbs J, Browne R, Saunders J, D'Souza R, Jungmann E. Healthcare professional experiences of the shift in integrated sexual health (ISH) service delivery as a result of the SARS-CoV-2 pandemic: What can we learn and where do we go from here? International Journal of STD and AIDS [Internet]. 2020;31(SUPPL 12):74–5.

Reason for exclusion: Ineligible study design

Gill H, Babatunde O, Weissman S. Social determinants of health and disparities in linkage to care among newly diagnosed HIV cases-South Carolina, 2009-2011. Open Forum Infectious Diseases [Internet]. 2017;4(Supplement 1):S419–20.

Reason for exclusion: Duplicate study

Gill H, Babatunde O, Weissman S. Social determinants of health and disparities in linkage to care among newly diagnosed HIV cases-South Carolina, 2009-2011. Open Forum Infectious Diseases [Internet]. 2017;4(Supplement 1):S419–20.

Reason for exclusion: Ineligible study design

Gimbel S, Mocumbi AO, Asbjornsdottir K, Coutinho J, Andela L, Cebola B, et al. Systems analysis and improvement approach to optimize the hypertension diagnosis and case cascade for PLHIV individuals (SAIA-HTN): a hybrid type III cluster randomized trial. Implementation science [Internet]. 2020;15(1).

Reason for exclusion: Ineligible phenomena of interest

Gimbel S, Rustagi AS, Robinson J, Kouyate S, Coutinho J, Nduati R, et al. Evaluation of a Systems Analysis and Improvement Approach to Optimize Prevention of Mother-To-Child Transmission of HIV Using the Consolidated Framework for Implementation Research. Journal of Acquired Immune Deficiency Syndromes: JAIDS [Internet]. 2016;72 Suppl 2:S108-16.

Reason for exclusion: Ineligible phenomena of interest

Gimbel S, Rustagi AS, Robinson J, Kouyate S, Coutinho J, Nduati R, et al. Evaluation of a Systems Analysis and Improvement Approach to Optimize Prevention of Mother-To-Child Transmission of HIV Using the Consolidated Framework for Implementation Research. Journal of Acquired Immune Deficiency Syndromes: JAIDS [Internet]. 2016;72 Suppl 2:S108-16.

Reason for exclusion: Ineligible phenomena of interest

Gimbel S, Voss J, Mercer MA, Zierler B, Gloyd S, Coutinho Mde J, et al. The prevention of mother-to-child transmission of HIV cascade analysis tool: supporting health managers to improve facility-level service delivery. BMC Research Notes [Internet]. 2014;7:743.

Reason for exclusion: Ineligible phenomena of interest

Gimbel S, Voss J, Rustagi A, Mercer MA, Zierler B, Gloyd S, et al. What does high and low have to do with it? Performance classification to identify health system factors associated with effective prevention of mother-to-child transmission of HIV delivery in Mozambique. Journal of the International AIDS Society [Internet]. 2014;17.

Reason for exclusion: Duplicate

Gipson JD, Uysal J, Narasimhan S, Gultiano S. Using Systematic Anomalous Case Analysis to Examine Sexual and Reproductive Health Outcomes in the Philippines. Studies in Family Planning [Internet]. 2020;51(2):139–59.

Reason for exclusion: Ineligible phenomena of interest

Gogishvili M, Florez KR, Costa SA, Huang TTK. A qualitative study on mixed experiences of discrimination and healthcare access among HIV-positive immigrants in Spain. BMC Public Health [Internet]. 2021;21(1).

Reason for exclusion: Ineligible phenomena of interest

Gonzales-Zamora JA, Ponce-Rosas L, Martinez R. Determinants of public health and interventions to address HIV infection among men who have sex with men in Miami-Dade County, Florida, USA. Infezioni in Medicina [Internet]. 2022;30(3):392–402.

Reason for exclusion: Ineligible study design

Govender K, Cowden RG, Asante KO, George G, Reardon C. Sexual Risk Behavior: a Multi-System Model of Risk and Protective Factors in South African Adolescents. Prevention Science [Internet]. 2019;20(7):1054–65.

Reason for exclusion: Ineligible phenomena of interest

Govender K, Seeley J, Watts C. Addressing structural factors in HIV prevention and treatment. African Journal of AIDS Research [Internet]. 2014;13(2):iii–v.

Reason for exclusion: Ineligible study design

Goyal M, Baskonus HM, Prakash A. Regarding new positive, bounded and convergent numerical solution of nonlinear time fractional HIV/AIDS transmission model. Chaos Solitons & Fractals [Internet]. 2020;139.

Reason for exclusion: Ineligible phenomena of interest

Green A, Kolar K. Engineering behaviour change in an epidemic: the epistemology of NIH-funded HIV prevention science. Sociology of Health & Illness [Internet]. 2015;37(4):561–77.

Reason for exclusion: Ineligible phenomena of interest

Greenhalgh T, Macfarlane F, Barton-Sweeney C, Woodard F. "If we build it, will it stay?" A case study of the sustainability of whole-system change in London. Milbank Quarterly [Internet]. 2012;90(3):516–47.

Reason for exclusion: Ineligible phenomena of interest

Grobbelaar SS, van der Merwe E. Supporting Inclusive Innovation: Developing Improved Analytical Methods and STI Policy Instruments to Operationalise Inclusive Innovation. In: Portland International Conference on Management of Engineering and Technology (PICMET) [Internet]. 2016. p. 90–102.

Reason for exclusion: Ineligible condition

GutiÃ©rrez MA, Quevedo MF, Valle SM, Jacques-AviÃ±Ã³ C, David ED, CaylÃ JA, et al. Acceptability and effectiveness of using mobile applications to promote HIV and other STI testing among men who have sex with men in Barcelona, Spain. Sexually Transmitted Infections. 2018;94(6):443–8.

Reason for exclusion: Ineligible phenomena of interest

Gwadz M, Leonard NR, Honig S, Freeman R, Kutnick A, Ritchie AS. Doing battle with "the monster:" how highrisk heterosexuals experience and successfully manage HIV stigma as a barrier to HIV testing. International Journal for Equity in Health [Internet]. 2018;17.

Reason for exclusion: Ineligible phenomena of interest

Gwadz M, de Guzman R, Freeman R, Kutnick A, Silverman E, Leonard NR, et al. Exploring How Substance Use Impedes Engagement along the HIV Care Continuum: A Qualitative Study. Frontiers in public health [Internet]. 2016;4.

Reason for exclusion: Ineligible phenomena of interest

Haas SM, Perazzo JD, Ruffner AH, Lyons MS. Exploring current stereotypes and norms impacting sexual partner HIV-status communication. Health Communication. 2020;35(11):1376–85.

Reason for exclusion: Ineligible phenomena of interest

Haberland N, Rogow D. Sexuality education: emerging trends in evidence and practice. Journal of Adolescent Health. 2015;56(1):S15–21.

Reason for exclusion: Ineligible study design

Hanvoravongchai P, Warakamin B, Coker R. Critical interactions between Global Fund-supported programmes and health systems: a case study in Thailand. Health policy and planning [Internet]. 2010;25 Suppl 1:i53-57.

Reason for exclusion: Ineligible phenomena of interest

Haregu TN, Setswe G, Elliott J, Oldenburg B. A tool to guide the process of integrating health system responses to public health problems. Healthcare in Low-Resource Settings [Internet]. 2015;3(1).

Reason for exclusion: Ineligible phenomena of interest

Haregu TN, Setswe G, Elliott J, Oldenburg B. Developing an action model for integration of health system response to HIV/AIDS and noncommunicable diseases (NCDs) in developing countries. Global Journal of Health Science [Internet]. 2013;6(1):9–22.

Reason for exclusion: Ineligible study design

Harinarain N, Haupt TC. Drivers for the effective management of HIV and AIDS in the South African construction industry - A Delphi study. African Journal of AIDS Research [Internet]. 2014;13(3):291–303.

Reason for exclusion: Duplicate study

Harinarain N, Haupt TC. Drivers for the effective management of HIV and AIDS in the South African construction industry--a Delphi study. African Journal of AIDS Research [Internet]. 2014;13(3):291–303.

Reason for exclusion: Ineligible phenomena of interest

Heijman T, Zuure F, Stolte I, Davidovich U. Motives and barriers to safer sex and regular STI testing among MSM soon after HIV diagnosis. BMC Infectious Diseases. 2017;17(1):1–11.

Reason for exclusion: Ineligible phenomena of interest

Herbst JH, Glassman M, Carey JW, Painter TM, Gelaude DJ, Fasula AM, et al. Operational research to improve HIV prevention in the United States. Journal of Acquired Immune Deficiency Syndromes: JAIDS [Internet]. 2012;59(5):530–6.

Reason for exclusion: Ineligible study design

Hernandez-Vargas EA, Colaneri P, Middleton RH. Optimal therapy scheduling for a simplified HIV infection model. Automatica [Internet]. 2013;49(9):2874–80.

Reason for exclusion: Ineligible context

Hernandez-Vargas EA, Middleton RH, Colaneri P, Blanchini F, Ieee. Dynamic Optimization Algorithms to mitigate HIV escape. In: 49th IEEE Conference on Decision and Control (CDC) [Internet]. 2010. p. 827–32.

Reason for exclusion: Ineligible context

Hightow-Weidman LB, Fowler B, Kibe J, McCoy R, Pike E, Calabria M, et al. HealthMpowerment. org: development of a theory-based HIV/STI website for young black MSM. AIDS education and prevention: official publication of the International Society for AIDS Education. 2011;23(1):1.

Reason for exclusion: Ineligible phenomena of interest

Holliday RC, Phillips R, Akintobi TH. A community-based participatory approach to the development and implementation of an HIV health behavior intervention: lessons learned in navigating research and practice systems from Project HAPPY. International Journal of Environmental Research and Public Health. 2020;17(2):399.

Reason for exclusion: Ineligible phenomena of interest

Holt M, Newman CE, Lancaster K, Smith AK, Hughes S, Truong HM. HIV preâ◎◎exposure prophylaxis and the â◎◎problemsâ◎◎ of reduced condom use and sexually transmitted infections in Australia: a critical analysis from an evidenceâ◎◎making intervention perspective. Sociology of Health & Illness. 2019;41(8):1535–48.

Reason for exclusion: Ineligible phenomena of interest

Horvath KJ, Bauermeister JA. eHealth literacy and intervention tailoring impacts the acceptability of a HIV/STI testing intervention and sexual decision making among young gay and bisexual men. AIDS education and prevention: official publication of the International Society for AIDS Education. 2017;29(1):14.

Reason for exclusion: Ineligible phenomena of interest

Hottes TS, Farrell J, Bondyra M, Haag D, Shoveller J, Gilbert M. Internet-based HIV and sexually transmitted infection testing in British Columbia, Canada: opinions and expectations of prospective clients. Journal of Medical Internet Research. 2012;14(2):e1948.

Reason for exclusion: Ineligible phenomena of interest

Hotton AL, French AL, Hosek SG, Kendrick SR, Lemos D, Brothers J, et al. Relationship dynamics and sexual risk reduction strategies among heterosexual young adults: A qualitative study of sexually transmitted infection clinic attendees at an urban Chicago health center. AIDS Patient Care and STDs. 2015;29(12):668–74.

Reason for exclusion: Ineligible phenomena of interest

Idowu PA. A Spatial Data Model for HIV/AIDS Surveillance and Monitoring in Nigeria. International Journal of E-Health and Medical Communications [Internet]. 2012;3(2):66–84.

Reason for exclusion: Ineligible phenomena of interest

Ingram MV, Amodei N, Perez VV, German V. Factors predicting 12-month retention in care for minority women living with HIV. Therapeutic Advances in Infectious Disease [Internet]. 2022;9:20499361221089812.

Reason for exclusion: Ineligible phenomena of interest

Iskarpatyoti BS, Lebov J, Hart L, Thomas J, Mandal M. Evaluations of Structural Interventions for HIV Prevention: A Review of Approaches and Methods. AIDS & Behavior [Internet]. 2018;22(4):1253–64.

Reason for exclusion: Ineligible study design

Jahn A, Harries AD, Schouten EJ, Libamba E, Ford N, Maher D, et al. Scaling-up antiretroviral therapy in Malawi. Bulletin of the World Health Organization [Internet]. 2016;94(10):772–6.

Reason for exclusion: Ineligible phenomena of interest

Jemmott LS, Jemmott JB, Lanier Y, Thompson C, Baker JL. Development of a barbershop-based HIV/STI risk reduction intervention for young heterosexual African American men. Health Promotion Practice. 2017;18(1):110–8.

Reason for exclusion: Ineligible phenomena of interest

Joore IK, van Roosmalen SL, van Bergen JE, van Dijk N. General practitionersâ©© barriers and facilitators towards new provider-initiated HIV testing strategies: a qualitative study. International Journal of STD & AIDS. 2017;28(5):459–66.

Reason for exclusion: Ineligible phenomena of interest

Kadia BM, Dimala CA, Njefi KP. Emergence of universal antiretroviral therapy coverage in South Africa: applying the advocacy coalition framework to refine the narratives and inform epidemic responses. The Pan African medical journal [Internet]. 2022;42:6.

Reason for exclusion: Ineligible study design

Kahn M. Mitigating South Africa's HIV Epidemic: The Interplay of Social Entrepreneurship and the Innovation System. Minerva [Internet]. 2016;54(2):129–50.

Reason for exclusion: Ineligible phenomena of interest

Karris MY, Beekmann SE, Mehta SR, Anderson CM, Polgreen PM. Are we prepped for preexposure prophylaxis (PrEP)? Provider opinions on the real-world use of PrEP in the United States and Canada. Clinical Infectious Diseases [Internet]. 2014;58(5):704–12.

Reason for exclusion: Ineligible phenomena of interest

Kassa SM. Three-level global resource allocation model for hiv control: A hierarchical decision system approach. Mathematical biosciences and engineering : MBE [Internet]. 2018;15(1):255–73.

Reason for exclusion: Ineligible phenomena of interest

Kerr CC, Stuart RM, Gray RT, Shattock AJ, Fraser-Hurt N, Benedikt C, et al. Optima: A Model for HIV Epidemic Analysis, Program Prioritization, and Resource Optimization. Journal of Acquired Immune Deficiency Syndromes: JAIDS [Internet]. 2015;69(3):365–76.

Reason for exclusion: Ineligible phenomena of interest

Kerrigan D, Kennedy CE, Morgan-Thomas R, Reza-Paul S, Mwangi P, Win KT, et al. A community empowerment approach to the HIV response among sex workers: effectiveness, challenges, and considerations for implementation and scale-up. The Lancet. 2015;385(9963):172–85.

Reason for exclusion: Ineligible study design

Keugoung B, Fotsing R, Macq J, Buve A, Marchal B, Meli J, et al. Does the National HIV/AIDS control programme provide support for district hospitals in Cameroon? Sante Publique [Internet]. 2015;27(4):547–56.

Reason for exclusion: Not English

Khan M, Golin C, Friedman S, Scheidell J, Adimora A, Judon-Monk S, et al. STI/HIV sexual risk behavior and prevalent STI among incarcerated African American men in committed partnerships: the significance of poverty, mood disorders, and substance use. AIDS and behavior. 2015;19(8):1478–90.

Reason for exclusion: Ineligible phenomena of interest

Khan MR, Scheidell JD, Rosen DL, Geller A, Brotman LM. Early age at childhood parental incarceration and STI/HIV-related drug use and sex risk across the young adult lifecourse in the US: Heightened vulnerability of black and Hispanic youth. Drug and Alcohol Dependence. 2018;183:231–9.

Reason for exclusion: Ineligible phenomena of interest

Kharfen M, Barnes C, Lago L, Dwyer G, Horton K, Seiler N. New Reimbursement and Integrated Planning: Policy Approaches to Reduce the Morbidity, Mortality, and Incidence of HIV in Washington, DC. Public Health Reports [Internet]. 2020;135(1_suppl):19S–24S.

Reason for exclusion: Ineligible study design

Kibicho J, Campbell JK. Community perspectives of second-generation alcohol misuse and HIV risk in rural Kenya: A gendered syndemic lens. Global Public Health [Internet]. 2019;14(12):1733–43.

Reason for exclusion: Ineligible phenomena of interest

Kiekens A, Bwire GM, Decouttere C, Jordan MR, Mangara A, Mosha IH, et al. HIV and SARS-CoV-2: the interplay of two wicked problems. BMJ global health [Internet]. 2022;7(8) (no pagination).

Reason for exclusion: Ineligible study design

Kouanda S, Nahyuha Chomi E, Kim C, Jen S, Bahamondes L, Cecatti JG, et al. Health systems analysis and evaluation of the barriers to availability, utilisation and readiness of sexual and reproductive health services in COVID-19-affected areas: a WHO mixed-methods study protocol. BMJ Open [Internet]. 2022;12(6):e057810.

Reason for exclusion: Ineligible phenomena of interest

Kumar K, Reyneke M, Du Toit T, Thompson D, Barday Z, Manning K, et al. A systems thinking approach: Barriers to implementation of an HIV-to-HIV positive transplant program in the state sector of South Africa. Transplantation [Internet]. 2018;102(7 Supplement 1):S561.

Reason for exclusion: Duplicate study

Kumar K, Reyneke M, Du Toit T, Thompson D, Barday Z, Manning K, et al. A systems thinking approach: Barriers to implementation of an HIV-to-HIV positive transplant program in the state sector of South Africa. Transplantation [Internet]. 2018;102(7 Supplement 1):S561.

Reason for exclusion: Ineligible study design

Kurth AE, Lally MA, Choko AT, Inwani IW, Fortenberry JD. HIV testing and linkage to services for youth. Journal of the International AIDS Society. 2015;18:19433.

Reason for exclusion: Ineligible study design

Lanham HJ, Leykum LK, Taylor BS, McCannon CJ, Lindberg C, Lester RT. How complexity science can inform scale-up and spread in health care: Understanding the role of self-organization in variation across local contexts. Social Science and Medicine [Internet]. 2013;93:194–202.

Reason for exclusion: Ineligible phenomena of interest

Latkin C, Weeks MR, Glasman L, Galletly C, Albarracin D. A dynamic social systems model

for considering structural factors in HIV prevention and detection. AIDS & Behavior [Internet]. 2010;14(Suppl 2):222–38.

Reason for exclusion: ineligible publication date

Lauby J, Milnamow M, Joseph HA, Hitchcock S, Carson L, Pan Y, et al. Evaluation of Project RISE, an HIV Prevention Intervention for Black Bisexual Men Using an Ecosystems Approach. AIDS & Behavior [Internet]. 2018;22(1):164–77.

Reason for exclusion: Ineligible phenomena of interest

Lea B, Deep K, Wilson J. A quality improvement intervention to increase HIV screening in primary care. Journal of Investigative Medicine [Internet]. 2010;58(2):501.

Reason for exclusion: Ineligible study design

Lebcir RM, Atun RA, Coker RJ. System Dynamic simulation of treatment policies to address colliding epidemics of tuberculosis, drug resistant tuberculosis and injecting drug users driven HIV in Russia. Journal of the Operational Research Society [Internet]. 2010;61(8):1238–48.

Reason for exclusion: ineligible publication date

Lee SS, Ho KM, Cheung GMT. The spatial context of clinic-reported sexually transmitted infection in Hong Kong. BMC Infectious Diseases [Internet]. 2010;10.

Reason for exclusion: Ineligible phenomena of interest

Lee Y, Dancy B, Florez E, Holm K. Factors related to sexual practices and successful sexually transmitted infection/HIV intervention programs for Latino adolescents. Public Health Nursing. 2013;30(5):390–401.

Reason for exclusion: Ineligible study design

Leerlooijer JN, Ruiter RA, Reinders J, Darwisyah W, Kok G, Bartholomew LK. The World Starts With Me: using intervention mapping for the systematic adaptation and transfer of school-based sexuality education from Uganda to Indonesia. Translational Behavioral Medicine [Internet]. 2011;1(2):331–40.

Reason for exclusion: Ineligible phenomena of interest

Lees S, Kielmann K, Cataldo F, Gitau-Mburu D. Understanding the linkages between informal and formal care for people living with HIV in sub-Saharan Africa. Global Public Health [Internet]. 2012;7(10):1109–19.

Reason for exclusion: Ineligible study design

Leston J, Tulloch S, Reilley B. Quality improvement: A systems approach to reducing health disparities. Sexually Transmitted Diseases [Internet]. 2014;1):S134.

Reason for exclusion: Ineligible condition

Leston J, Tulloch S, Reilley B. Quality improvement: A systems approach to reducing health disparities. Sexually Transmitted Diseases [Internet]. 2014;1):S134.

Reason for exclusion: Ineligible study design

Leston JD, Jessen CM, Simons BC. Alaska Native and Rural Youths' Views of Sexual Health: A Focus Group Project on Sexually Transmitted Diseases, HIV/AIDS, and Unplanned Pregnancy. American Indian and Alaska Native Mental Health Research: The Journal of the National Center. 2012;19(1):1–14.

Reason for exclusion: Ineligible phenomena of interest

Levy ME, Wilton L, Phillips G, Glick SN, Kuo I, Brewer RA, et al. Understanding structural barriers to accessing HIV testing and prevention services among black men who have sex with men (BMSM) in the United States. AIDS & Behavior [Internet]. 2014;18(5):972–96.

Reason for exclusion: Ineligible study design

Lewis KM, Lesesne CA, Zahniser SC, Wilson MM, Desiderio G, Wandersman A, et al. Developing a prevention synthesis and translation system to promote science-based approaches to teen pregnancy, HIV and STI prevention. American Journal of Community Psychology. 2012;50:553–71.

Reason for exclusion: Ineligible study design

Li DH, Brown CH, Gallo C, Morgan E, Sullivan PS, Young SD, et al. Design Considerations for Implementing eHealth Behavioral Interventions for HIV Prevention in Evolving Sociotechnical Landscapes. Current HIV/AIDS Reports [Internet]. 2019;16(4):335–48.

Reason for exclusion: Ineligible phenomena of interest

Li MN, Yu WY, Tian W, Ge Y, Liu Y, Ding T, et al. System dynamics modeling of public health services provided by China CDC to control infectious and endemic diseases in China. Infection and Drug Resistance [Internet]. 2019;12:613–25.

Reason for exclusion: Ineligible condition

Li YH, Mgbere O, Abughosh S, Chen H, Cuccaro P, Smesny A, et al. Assessment of sexually transmitted disease/HIV risk among young African Americans: comparison of self-perceived and epidemiological risks utilizing ecodevelopmental theory. Hiv Aids-Research and Palliative Care [Internet]. 2019;11:31–44.

Reason for exclusion: Ineligible phenomena of interest

Lillie TA, Baer J, Adams D, Zhao J, Wolf RC. Think global, act local: the experience of Global Fund and PEPFAR joint cascade assessments to harmonize and strengthen key population HIV programmes in eight countries. Journal of the International AIDS Society. 2018;21:e25125.

Reason for exclusion: Ineligible study design

Lima MCL, Pinho CM, Dourado CARO, Silva MASD, Andrade MS. Diagnostic aspects and in-service training in the decentralization of care to people living with HIV. Revista da Escola de Enfermagem da U S P [Internet]. 2021;55:e20210065.

Reason for exclusion: Ineligible phenomena of interest

Logie CH, Okumu M, Kibuuka Musoke D, Hakiza R, Mwima S, Kyambadde P, et al. Intersecting stigma and HIV testing practices among urban refugee adolescents and youth in Kampala, Uganda: qualitative findings. Journal of the International AIDS Society. 2021;24(3):e25674.

Reason for exclusion: Ineligible phenomena of interest

Lorway R, Shaw SY, Hwang SD, Reza-Paul S, Pasha A, Wylie JL, et al. From individuals to complex systems: exploring the sexual networks of men who have sex with men in three cities of Karnataka, India. Sexually Transmitted Infections [Internet]. 2010;86 Suppl 3:iii70-78.

Reason for exclusion: ineligible publication date

Lowenthal ED, DeLong SM, Zanoni B, Njuguna I, Beima-Sofie K, Dow D, et al. Impact of COVID-19 on Adolescent HIV Prevention and Treatment Research in the AHISA Network. AIDS & Behavior [Internet]. 2022;12:12.

Reason for exclusion: Ineligible phenomena of interest

Lucky TT, McDonald AM, Wand H, Lam M, El-Hayek C, Wilson DP. Merging Australia's national HIV and AIDS registries: improving quality and completeness of data. Australian & New Zealand Journal of Public Health [Internet]. 2013;37(4):393–4.

Reason for exclusion: Duplicate study

Lunsford SS, Fatta K, Stover KE, Shrestha R. Supporting close-to-community providers through a community health system approach: case examples from Ethiopia and Tanzania. Human resources for health [Internet]. 2015;13:12.

Reason for exclusion: Ineligible phenomena of interest

Lutete P, Matthews DW, Sabounchi NS, Paige MQ, Lounsbury DW, Rodriguez N, et al. Intersectional Stigma and Prevention Among Gay, Bisexual, and Same Gender-Loving Men in New York City, 2020: System Dynamics Models. American Journal of Public Health [Internet]. 2022;112(S4):S444–51.

Reason for exclusion: Duplicate study

Luwanda AG, Mwambi HG. A Nonlinear Mixed-Effects Model for Multivariate Longitudinal Data with Dropout with Application to HIV Disease Dynamics. Journal of Agricultural Biological and Environmental Statistics [Internet]. 2016;21(2):277–94.

Reason for exclusion: Ineligible phenomena of interest

MacGregor H, McKenzie A, Jacobs T, Ullauri A. Scaling up ART adherence clubs in the public sector health system in the Western Cape, South Africa: a study of the institutionalisation of a pilot innovation. Global Health [Internet]. 2018;14(1):40.

Reason for exclusion: Ineligible phenomena of interest

MacPherson P, Van Rooyen H, Mukoma W, Chepuka L, Tulloch O, Baggaley R, et al. Risks and benefits of the introduction of HIV self-testing in the UK: Lessons from Kenya, Malawi and South Africa to inform a public health approach. HIV Medicine [Internet]. 2014;3):99–

100.

Reason for exclusion: Ineligible study design

Maher D. Re-thinking global health sector efforts for HIV and tuberculosis epidemic control: promoting integration of programme activities within a strengthened health system. BMC Public Health [Internet]. 2010;10:394.

Reason for exclusion: Ineligible study design

Makusha T, Knight L, Taegtmeyer M, Tulloch O, Davids A, Lim J, et al. HIV self-testing could â◎◎revolutionize testing in South Africa, but it has got to be done properlyâ◎◎: perceptions of key stakeholders. PLoS ONE. 2015;10(3):e0122783.

Reason for exclusion: Ineligible phenomena of interest

Markham CM, Rushing SC, Manthei J, Singer M, Jessen C, Gorman G, et al. The Healthy Native Youth Implementation Toolbox: Using Implementation Mapping to adapt an online decision support system to promote culturally-relevant sexual health education for American Indian and Alaska Native youth. Frontiers in public health [Internet]. 2022;10:889924.

Reason for exclusion: Ineligible phenomena of interest

Marshall B, Paczkowski M, Tempalski B, Pouget E, Friedman S, Galea S. Combination interventions for the prevention of HIV among injection drug users: A complex systems dynamics model. Journal of the International AIDS Society [Internet]. 2012;3):108.

Reason for exclusion: Ineligible study design

Martinez O, Lopez N, Woodard T, Rodriguez-Madera S, Icard L. Transhealth information project: a peer-led HIV prevention intervention to promote HIV protection for individuals of transgender experience. Health & Social Work. 2019;44(2):104–12.

Reason for exclusion: Ineligible phenomena of interest

Martinez O, Wu E, Frasca T, Shultz AZ, Fernandez MI, Rios JL, et al. Adaptation of a Couple-Based HIV/STI Prevention Intervention for Latino Men Who Have Sex With Men in New York City. American Journal of Mens Health [Internet]. 2017;11(2):181–95.

Reason for exclusion: Duplicate study

Martinez O, Wu E, Levine EC, MuÃ±oz-Laboy M, Fernandez MI, Bass SB, et al. Integration of social, cultural, and biomedical strategies into an existing couple-based behavioral HIV/STI prevention intervention: voices of Latino male couples. PLoS ONE. 2016;11(3):e0152361.

Reason for exclusion: Ineligible phenomena of interest

Marum E, Taegtmeyer M, Parekh B, Mugo N, Lembariti S, Phiri M, et al. "What Took You So Long?" The Impact of PEPFAR on the Expansion of HIV Testing and Counseling Services in Africa. Jaids-Journal of Acquired Immune Deficiency Syndromes [Internet]. 2012;60:S63–9.

Reason for exclusion: Ineligible study design

Matson PA, Wilkinson A, Lich KH, Ellen JM, Anderson SL. Using a systems approach to design higherimpact interventions: Illustrating levels of leverage for reducing concurrency-linked sexually transmitted infections in African Amercian adolescents from disadvantaged communities. Sexually Transmitted Infections [Internet]. 2017;93(Supplement 2):A219–20.

Reason for exclusion: Ineligible study design

Mayhew SH, Warren CE, Ndwiga C, Narasimhan M, Wilcher R, Mutemwa R, et al. Health systems software factors and their effect on the integration of sexual and reproductive health and HIV services. The lancet HIV [Internet]. 2020;7(10):e711–20.

Reason for exclusion: Ineligible study design

Mburu G, Oxenham D, Hodgson I, Nakiyemba A, Seeley J, Bermejo A. Community systems strengthening for HIV care: experiences from Uganda. Journal Of Social Work In End-Of-Life & Palliative Care [Internet]. 2013;9(4):343–68.

Reason for exclusion: Ineligible phenomena of interest

McCombe G, Murtagh S, Lazarus JV, Van Hout MC, Bachmann M, Jaffar S, et al. Integrating diabetes, hypertension and HIV care in sub-Saharan Africa: a Delphi consensus study on international best practice. BMC Health Services Research [Internet]. 2021;21(1):1235.

Reason for exclusion: Ineligible phenomena of interest

McInroy LB, Hawkins BW, Zapcic I, Fregoli C. Design Thinking for Health Disparities and Interdisciplinary Knowledge Translation: An LGBTQ+ Youth Health Literacy Project. Health & Social Work [Internet]. 2022;1:1.

Reason for exclusion: Ineligible phenomena of interest

McKay VR, Margaret Dolcini M, Hoffer LD. The dynamics of de-adoption: a case study of policy change, de-adoption, and replacement of an evidence-based HIV intervention. Translational Behavioral Medicine [Internet]. 2017;7(4):821–31.

Reason for exclusion: Ineligible phenomena of interest

Medeiros P. Mapping HIV-related services for women in Eastern Canada: A qualitative study. Womens Health [Internet]. 2022;18.

Reason for exclusion: Ineligible phenomena of interest

Mgbere O, Monjok E, Abughosh S, Ekong E, Holstad MM, Essien EJ. Modeling covariates of self-perceived and epidemiologic notions of risk for acquiring STIs/HIV among military personnel: a comparative analysis. AIDS and behavior. 2013;17(3):1159–75.

Reason for exclusion: Ineligible phenomena of interest

Millard T, Dodson S, McDonald K, Klassen KM, Osborne RH, Battersby MW, et al. The systematic development of a complex intervention: HealthMap, an online self-management support program for people with HIV. BMC Infectious Diseases [Internet]. 2018;18(1) (no pagination).

Reason for exclusion: Ineligible phenomena of interest

Miller RL, Levine RL, McNall MA, Khamarko K, Valenti MT. A dynamic model of client recruitment and retention in community-based HIV prevention programs. Health Promotion Practice [Internet]. 2011;12(1):135–46.

Reason for exclusion: ineligible publication date

Mishra A, Garner A. A whole systems approach to improving HIV testing in UK general practice. HIV Medicine [Internet]. 2022;23:68–9.

Reason for exclusion: Ineligible study design

Mora C, Monteiro S. Vulnerability to STIs/HIV: sociability and the life trajectories of young women who have sex with women in Rio de Janeiro. Culture, Health & Sexuality. 2010;12(1):115–24.

Reason for exclusion: Ineligible phenomena of interest

Morales A, Vallejo-Medina P, Abello-Luque D, Saavedra-Roa A, GarcÃ-a-Roncallo P, Gomez-Lugo M, et al. Sexual risk among Colombian adolescents: knowledge, attitudes, normative beliefs, perceived control, intention, and sexual behavior. BMC Public Health. 2018;18(1):1–13.

Reason for exclusion: Ineligible phenomena of interest

Moreira C, Boughey A, Ryan KE, Higgins N, Rotty J, West M, et al. Two decades of surveillance data show late presentation among a diverse group of women diagnosed with HIV in Victoria, Australia. Australian and New Zealand journal of public health [Internet]. 2019;43(5):413–8.

Reason for exclusion: Ineligible phenomena of interest

Mugassa AM, Frumence G. Factors influencing the uptake of cervical cancer screening services in Tanzania: A health system perspective from national and district levels. Nursing Open [Internet]. 2020;7(1):345–54.

Reason for exclusion: Ineligible condition

Mugavero MJ. Elements of the HIV Care Continuum: Improving Engagement and Retention in Care. Topics in antiviral medicine [Internet]. 2016;24(3):115–9.

Reason for exclusion: Ineligible phenomena of interest

Munro-Kramer ML, Fava NM, Banerjee T, Darling-Fisher CS, Pardee M, Villarruel AM, et al. The Effect of a Youth-Centered Sexual Risk Event History Calendar (SREHC) Assessment on Sexual Risk Attitudes, Intentions, and Behavior. Journal of pediatric health care [Internet]. 2017;31(3):302â◎◎313.

Reason for exclusion: Ineligible phenomena of interest

Mustanski B, Garofalo R, Monahan C, Gratzer B, Andrews R. Feasibility, acceptability, and preliminary efficacy of an online HIV prevention program for diverse young men who have sex with men: the keep it up! intervention. AIDS and behavior. 2013;17(9):2999–3012.

Reason for exclusion: Ineligible phenomena of interest

Nattabi B, Girgis S, Matthews V, Bailie R, Ward JE. Clinic predictors of better syphilis testing in Aboriginal primary healthcare: a promising opportunity for primary healthcare service managers. Australian Journal of Primary Health [Internet]. 2018;24(4):350–8.

Reason for exclusion: Ineligible phenomena of interest

Nattrass N. Strengthening health systems Perspective paper [Internet]. Rethinkhiv: Smarter Ways to Invest in Ending Hiv in Sub-Saharan Africa. 2012.

Reason for exclusion: Ineligible study design

Nct. Incentives for HIV Testing in Women. https://clinicaltrials.gov/show/NCT03768986 [Internet]. 2018;

Reason for exclusion: Ineligible phenomena of interest

Nct. Scaling up TB and HIV Treatment Integration. https://clinicaltrials.gov/show/NCT02654613 [Internet]. 2016;

Reason for exclusion: Ineligible study design

Nct. Testing an Intervention to Increase HIV Self-Testing Among Young, Black MSM. https://clinicaltrials.gov/show/NCT04210271 [Internet]. 2019;

Reason for exclusion: Ineligible study design

Nct. We Are Here Now: a Multi-level, Multicomponent Sexual and Reproductive Health Intervention for American Indian Youth. https://clinicaltrials.gov/show/NCT03694418 [Internet]. 2018;

Reason for exclusion: Ineligible phenomena of interest

Newman PA, Guta A, Lacombeâ◎◎Duncan A, Tepjan S. Clinical exigencies, psychosocial realities: negotiating HIV preâ◎◎exposure prophylaxis beyond the cascade among gay, bisexual and other men who have sex with men in Canada. Journal of the International AIDS Society. 2018;21(11):e25211.

Reason for exclusion: Ineligible phenomena of interest

Noar SM, Webb EM, Van Stee SK, Redding CA, Feist-Price S, Crosby R, et al. Using computer technology for HIV prevention among African-Americans: development of a tailored information program for safer sex (TIPSS). Health Education Research. 2011;26(3):393–406.

Reason for exclusion: ineligible publication date

Olakunde BO, Adeyinka DA, Olawepo JO, Pharr JR, Ozigbu CE, Wakdok S, et al. Towards the elimination of mother-to-child transmission of HIV in Nigeria: a health system perspective of the achievements and challenges. International Health [Internet]. 2019;11(4):240–9.

Reason for exclusion: Ineligible phenomena of interest

Ologhobo T, Middleton D, Opdyke KM. Analysis of capacity to bill for STD-related services in STD clinics that receive 340-B pricing in NJ and NYS using a public health systems approach. Sexually Transmitted Diseases [Internet]. 2014;1):S130.

Reason for exclusion: Ineligible study design

Omori R, Nagelkerke N, Abu-Raddad LJ. Nonpaternity and Half-Siblingships as Objective Measures of Extramarital Sex: Mathematical Modeling and Simulations. BioMed Research International [Internet]. 2017;2017:3564861.

Reason for exclusion: Ineligible condition

Opara I, Lizarraga A, Lardier DT, Herrera A, Garcia-Reid P, Reid RJ. What happens when we ask? A phenomenological focus group on HIV prevention and sexual health education among emancipated foster care youth. Children and Youth Services Review. 2022;140:106583.

Reason for exclusion: Ineligible phenomena of interest

Opara I. Examining African American Parent-Daughter HIV Risk Communication Using a Black Feminist-Ecological Lens: Implications for Intervention. Journal of Black Studies [Internet]. 2018;49(2):134–51.

Reason for exclusion: Ineligible phenomena of interest

Operario D, Nemoto T. HIV in transgender communities: syndemic dynamics and a need for multicomponent interventions. Journal of acquired immune deficiency syndromes (1999). 2010;55(Suppl 2):S91.

Reason for exclusion: Duplicate study

Operario D, Nemoto T. HIV in transgender communities: syndemic dynamics and a need for multicomponent interventions. Journal of acquired immune deficiency syndromes (1999). 2010;55(Suppl 2):S91.

Reason for exclusion: Ineligible study design

Opondo JO. The IDU continuum of care: Bringing together a Range of services for injection drug users (IDUS) in saskatoon health region (SHR) “making it Happen.” Canadian Journal of Infectious Diseases and Medical Microbiology [Internet]. 2010;SB):87B.

Reason for exclusion: Ineligible study design

Orellana ER, Alva IE, CÃ¡rcamo CP, GarcÃ-a PJ. Structural factors that increase HIV/STI vulnerability among indigenous people in the Peruvian amazon. Qualitative Health Research. 2013;23(9):1240–50.

Reason for exclusion: Ineligible phenomena of interest

Otis J, Martel M, Haig TA, Monteith K, Rousseau R, Herrera A. Understanding access to prevention strategies: Taking the first steps toward combination HIV prevention for MSM in Montreal. Canadian Journal of Infectious Diseases and Medical Microbiology [Internet]. 2015;SB):106B.

Reason for exclusion: Ineligible study design

Owens C, Hubach RD, Williams D, Voorheis E, Lester J, Reece M, et al. Facilitators and Barriers of Pre-exposure Prophylaxis (PrEP) Uptake Among Rural Men who have Sex with Men Living in the Midwestern US. Archives of Sexual Behavior [Internet]. 2020;49(6):2179–91.

Reason for exclusion: Ineligible phenomena of interest

O'Connell KA, Kisteneff AV, Gill SS, Edwards JF, Sherrerd-Smith WW, Moraczewski LA, et al. HIV post-exposure prophylaxis in the emergency department: An updated assessment and opportunities for HIV prevention identified. American Journal of Emergency Medicine [Internet]. 2021;46:323–8.

Reason for exclusion: Ineligible phenomena of interest

Pantalone DW, Holloway IW, Goldblatt AE, Gorman KR, Herbitter C, Grov C. The impact of pre-exposure prophylaxis on sexual communication and sexual behavior of urban gay and bisexual men. Archives of Sexual Behavior. 2020;49(1):147–60.

Reason for exclusion: Ineligible phenomena of interest

Patenaude B. Do changes in development assistance for health crowd out domestic investment for health and what are the implications for HIV/AIDS outcomes. Journal of the International AIDS Society Conference: 10th IAS Conference on HIV Science Mexico City Mexico [Internet]. 2019;22(Supplement 5).

Reason for exclusion: Ineligible study design

Penn C, Watermeyer J, Evans M. Why don't patients take their drugs? The role of communication, context and culture in patient adherence and the work of the pharmacist in HIV/AIDS. Patient Education and Counseling [Internet]. 2011;83(3):310–8.

Reason for exclusion: Ineligible phenomena of interest

Philips M, Cartier N, Dethier T, Akerfeldt K, Bemelmans M. Why do low HIV prevalence contexts in Africa often fail to reach effective ART coverage? Lessons learned on programmatic and delivery model adaptations in Guinea and Democratic Republic of Congo. Tropical Medicine and International Health [Internet]. 2013;1):177–8.

Reason for exclusion: Ineligible study design

Phillips AN, Bansi-Matharu L, Cambiano V, Ehrenkranz P, Serenata C, Venter F, et al. The potential role of long-acting injectable cabotegravir-rilpivirine in the treatment of HIV in sub-Saharan Africa: a modelling analysis. The Lancet Global Health [Internet]. 2021;9(5):e620–7.

Reason for exclusion: Ineligible phenomena of interest

Platteau T, Wouters K, Apers L, Avonts D, NÃ¶stlinger C, Sergeant M, et al. Voluntary outreach counselling and testing for HIV and STI among men who have sex with men in Antwerp. acta clinica Belgica. 2012;67(3):172–6.

Reason for exclusion: Ineligible phenomena of interest

Pulerwitz J, Michaelis A, Verma R, Weiss E. Addressing gender dynamics and engaging men in HIV programs: lessons learned from Horizons research. Public Health Reports. 2010;125(2):282–92.

Reason for exclusion: Ineligible phenomena of interest

Raftery AE, Bao L. Estimating and Projecting Trends in HIV/AIDS Generalized Epidemics Using Incremental Mixture Importance Sampling. Biometrics [Internet]. 2010;66(4):1162–73.

Reason for exclusion: Ineligible phenomena of interest

Ramanaik S, Thompson LH, du Plessis E, Pelto P, Annigeri V, Doddamane M, et al. Intimate relationships of Devadasi sex workers in South India: An exploration of risks of HIV/STI transmission. Global Public Health. 2014;9(10):1198–210.

Reason for exclusion: Ineligible phenomena of interest

Rand JR. Inuit women's stories of strength: informing Inuit community-based HIV and STI prevention and sexual health promotion programming. International journal of circumpolar health. 2016;75(1):32135.

Reason for exclusion: Ineligible phenomena of interest

Reid SE, Topp SM, Turnbull ER, Hatwiinda S, Harris JB, Maggard KR, et al. Tuberculosis and HIV control in sub-Saharan African prisons: thinking outside the prison cell. Journal of Infectious Diseases. 2012;205(suppl_2):S265–73.

Reason for exclusion: Ineligible phenomena of interest

Reid SE, Topp SM, Turnbull ER, Hatwiinda S, Harris JB, Maggard KR, et al. Tuberculosis and HIV control in sub-Saharan African prisons: thinking outside the prison cell. Journal of Infectious Diseases. 2012;205(suppl_2):S265–73.

Reason for exclusion: Duplicate

Reynolds EK, Magidson JF, Bornovalova MA, Gwadz M, Ewart CK, Daughters SB, et al. Application of the social action theory to understand factors associated with risky sexual behavior among individuals in residential substance abuse treatment. Psychology of Addictive Behaviors [Internet]. 2010;24(2):311–21.

Reason for exclusion: Ineligible phenomena of interest

Rink E, Anastario M, Johnson O, GrowingThunder R, Ricker A, Firemoon P, et al. The development and testing of a multi-level, multi-component pilot intervention to reduce sexual and reproductive health disparities in a tribal community. Journal of Ethnic & Cultural Diversity in Social Work [Internet]. 2021;30(1–2):138–48.

Reason for exclusion: Ineligible phenomena of interest

Rink E, Anastario M, Reimer GA, Peterson M. An ecological approach to understanding Women's reproductive health and pregnancy decision making in Greenland. Health & Place [Internet]. 2022;77:102868.

Reason for exclusion: Ineligible phenomena of interest

Rink E, Firemoon P, Anastario M, Johnson O, GrowingThunder R, Ricker A, et al. Rationale, Design, and Methods for Nen Unkumbi/Edahiyedo (“We Are Here Now”): a Multi-Level Randomized Controlled Trial to Improve Sexual and Reproductive Health Outcomes in a Northern Plains American Indian Reservation Community. Frontiers in public health [Internet]. 2022;10:823228.

Reason for exclusion: Ineligible phenomena of interest

Rios-Hincapie CY, Rojas M, Lopez M, Porras A, Luque R, Pelissari DM, et al. Delays in HIV and TB diagnosis and treatment initiation in co-infected patients in Colombia. International Journal of STD & AIDS [Internet]. 2020;31(5):410–9.

Reason for exclusion: Ineligible phenomena of interest

Risbud AR, Deshpande GR, Narayanan P, Parimi P, Das A. Neisseria gonorrhoea and chlamydia trachomatis re-infection and associated risk factors among cohort of female sex workers in India. Sexually Transmitted Infections [Internet]. 2011;1):A128.

Reason for exclusion: ineligible publication date

Riss I. Wave Pattern of Multi-Agent Social Actions. Systemic Practice and Action Research [Internet]. 9999; Available from: <Go to ISI>://WOS:000821970700001

Reason for exclusion: Ineligible outcomes

Rogstad K, Briggs A. Evaluating the effects of improvement initiatives in sexual health and HIV services using management theory, run charts and learning histories-an example from the introduction of electronic patient records. HIV Medicine [Internet]. 2014;3):33.

Reason for exclusion: Ineligible study design

Romero-Severson EO, Volz E, Koopman JS, Leitner T, Ionides EL. Dynamic Variation in Sexual Contact Rates in a Cohort of HIV-Negative Gay Men. American Journal of Epidemiology [Internet]. 2015;182(3):255–62.

Reason for exclusion: Ineligible phenomena of interest

Rudge JW, Phuanakoonon S, Nema KH, Mounier-Jack S, Coker R. Critical interactions between Global Fund-supported programmes and health systems: a case study in Papua New Guinea. Health policy and planning [Internet]. 2010;25 Suppl 1:i48-52.

Reason for exclusion: Ineligible phenomena of interest

SabidÃ³ M, Gregg LP, VallÃ¨s X, Nikiforov M, MonzÃ³n JE, Pedroza MI, et al. Notification for sexually transmitted infections and HIV among sex workers in Guatemala: acceptability, barriers, and preferences. Sexually Transmitted Diseases. 2012;504–8.

Reason for exclusion: Ineligible phenomena of interest

Sales J, DiClemente R, Davis T, Sullivan S. Exploring why young African American women do not change condom-use behavior following participation in an STI/HIV prevention intervention. Health Education Research. 2012;27(6):1091–101.

Reason for exclusion: Ineligible phenomena of interest

Sassman R, Lehaney B, Bali RK, Naguib RNG, Marshall IM. The Development of a Framework to Evaluate the Management of HIV/AIDS Programmes in Rural and Urban South Africa [Internet]. Perspectives of Knowledge Management in Urban Health. 2010.

Reason for exclusion: Ineligible outcomes

Scheibe A, Shelly S, Lambert A, Schneider A, Basson R, Medeiros N, et al. Using a programmatic mapping approach to plan for HIV prevention and harm reduction interventions for people who inject drugs in three South African cities. Harm Reduction Journal [Internet]. 2017;14(1):35.

Reason for exclusion: Ineligible phenomena of interest

Scheim AI, Travers R. Barriers and facilitators to HIV and sexually transmitted infections testing for gay, bisexual, and other transgender men who have sex with men. AIDS Care. 2017;29(8):990–5.

Reason for exclusion: Ineligible phenomena of interest

Schnall R, Rojas M, Travers J, Brown W, Bakken S. Use of Design Science for Informing the Development of a Mobile App for Persons Living with HIV. AMIA . Annual Symposium Proceedings/AMIA Symposium [Internet]. 2014;2014:1037–45.

Reason for exclusion: Ineligible phenomena of interest

Shannon K, Goldenberg SM, Deering KN, Strathdee SA. HIV infection among female sex workers in concentrated and high prevalence epidemics: why a structural determinants framework is needed. Current Opinion in HIV and AIDS. 2014;9(2):174.

Reason for exclusion: Ineligible study design

Sharma A, Kahle E, Todd K, Peitzmeier S, Stephenson R. Variations in testing for HIV and other sexually transmitted infections across gender identity among transgender youth. Transgender Health. 2019;4(1):46–57.

Reason for exclusion: Ineligible phenomena of interest

Sherr K, Silvis Rustagi A, Gimbel S, Nduati R, Cuembelo F, Kone A, et al. Impact of a systems engineering intervention on PMTCT service delivery in Côte d'Ivoire, Kenya, Mozambique: the SAIA cluster randomized trial. Journal of the International AIDS Society [Internet]. 2016;19:26.

Reason for exclusion: Ineligible phenomena of interest

Sherr K, Ásbjörnsdóttir K, Crocker J, Coutinho J, de Fatima Cuembelo M, Tavede E, et al. Scaling-up the Systems Analysis and Improvement Approach for prevention of mother-to-child HIV transmission in Mozambique (SAIA-SCALE): a stepped-wedge cluster randomized trial. Implementation science [Internet]. 2019;14(1):41.

Reason for exclusion: Ineligible phenomena of interest

Siegler AJ, Mbwambo JK, DiClemente RJ. Applying the Dynamic Social Systems Model to

HIV prevention in a rural African context: the Maasai and the esoto dance. Health Education & Behavior [Internet]. 2013;40(6):683–93.

Reason for exclusion: Ineligible phenomena of interest

Simone J, Hoyt MJ, Storm DS, Finocchario-Kessler S. Models of HIV Preconception Care and Key Elements Influencing These Services: Findings from Healthcare Providers in Seven US Cities. AIDS Patient Care and STDs [Internet]. 2018;32(7):272–81.

Reason for exclusion: Ineligible phenomena of interest

Siregar KN, Hanifah L, Rikawarastuti, Wahyuniar L. Prevention of HIV Transmission from Mother to Child: Challenges to the Successful Program Implementation and Practice in Indonesia. Journal of the International Association of Providers of AIDS Care [Internet]. 2021;20:23259582211040700.

Reason for exclusion: Ineligible phenomena of interest

Slinkard MS, Kazer MW. Older adults and HIV and STI screening: the patient perspective. Geriatric Nursing. 2011;32(5):341–9.

Reason for exclusion: Ineligible phenomena of interest

Smith RJ, Okano JT, Kahn JS, Bodine EN, Blower S. Evolutionary dynamics of complex networks of HIV drug-resistant strains: the case of San Francisco. Science [Internet]. 2010;327(5966):697–701.

Reason for exclusion: ineligible publication date

Song RG, Green TA, Hall HI. A Surveillance Data-Based Model System for Assessing the Effects of HIV Intervention and Prevention Strategies. Journal of Public Health Management and Practice [Internet]. 2021;27(2):E61–70.

Reason for exclusion: Ineligible phenomena of interest

Spadafino JT, Martinez O, Levine EC, Dodge B, MuÃ±oz-Laboy M, Fernandez MI. Correlates of HIV and STI testing among Latino men who have sex with men in New York City. AIDS Care. 2016;28(6):695–8.

Reason for exclusion: Ineligible phenomena of interest

Spicer N, Bogdan D, Brugha R, Harmer A, Murzalieva G, Semigina T. "It"s risky to walk in the city with syringes': understanding access to HIV/AIDS services for injecting drug users in the former Soviet Union countries of Ukraine and Kyrgyzstan. Globalization and Health [Internet]. 2011;7.

Reason for exclusion: ineligible publication date

Stahl JE, McGowan H, DiResta E, Gaydos CA, Klapperich C, Parrish J, et al. Systems Engineering and Point of Care Testing: Report from the NIBIB POCT/Systems Engineering Workshop. Point of Care: The Journal of Near-Patient Testing & Technology [Internet]. 2015;14(1):12–24.

Reason for exclusion: Ineligible phenomena of interest

Stahlman S, Grosso A, Ketende S, Sweitzer S, Mothopeng T, Taruberekera N, et al. Depression and social stigma among MSM in Lesotho: implications for HIV and sexually transmitted infection prevention. AIDS and behavior. 2015;19(8):1460–9.

Reason for exclusion: Ineligible phenomena of interest

Sullivan PS, Peterson J, Rosenberg ES, Kelley CF, Cooper H, Vaughan A, et al. Understanding racial HIV/STI disparities in black and white men who have sex with men: a multilevel approach. PLoS ONE. 2014;9(3):e90514.

Reason for exclusion: Ineligible phenomena of interest

Supervie V, Garcia-Lerma JG, Heneine W, Blower S. HIV, transmitted drug resistance, and the paradox of preexposure prophylaxis. Proceedings of the National Academy of Sciences of the United States of America [Internet]. 2010;107(27):12381–6.

Reason for exclusion: ineligible publication date

Sychareun V, Thomsen S, Chaleunvong K, Faxelid E. Risk perceptions of STIs/HIV and sexual risk behaviours among sexually experienced adolescents in the Northern part of Lao PDR. BMC Public Health. 2013;13(1):1–13.

Reason for exclusion: Ineligible phenomena of interest

Sznajder KK, Winchester MS, Biney AAE, Dodoo ND, Letsa D, Dodoo FNA. The Migration Experience and Differential Risks to Sexual and Reproductive Health in Ghana. Health education & behavior : the official publication of the Society for Public Health Education [Internet]. 2020;47(5):718–27.

Reason for exclusion: Ineligible phenomena of interest

Tafesse M. Investigating the Role of Media Advocacy in Framing Accessibility of Antiretroviral Treatment in Ethiopia: A Social Work Perspective on Communication Theories. Journal of Hiv-Aids & Social Services [Internet]. 2011;10(2):174–93.

Reason for exclusion: ineligible publication date

Tameru B, Habtemariam T, Nganwa D, Gerbi G, Bogale A, Robnett V, et al. Assessing HIV/AIDS intervention strategies using an integrative macro-micro level computational epidemiologic modeling approach. Ethnicity & Disease [Internet]. 2010;20(1 Suppl 1):S1-207–10.

Reason for exclusion: Ineligible phenomena of interest

Tan RKJ, Kaur N, Kumar PA, Tay E, Leong A, Chen MI-C, et al. Clinics as spaces of costly disclosure: HIV/STI testing and anticipated stigma among gay, bisexual and queer men. Culture, Health & Sexuality. 2020;22(3):307–20.

Reason for exclusion: Ineligible phenomena of interest

Theunissen KA, Hoebe CJ, Crutzen R, Kara-Zaitri C, de Vries NK, van Bergen JE, et al. Using intervention mapping for the development of a targeted secure web-based outreach strategy

named SafeFriend, for Chlamydia trachomatis testing in young people at risk. BMC Public Health [Internet]. 2013;13:996.

Reason for exclusion: Ineligible phenomena of interest

Thomas D, Wanje G, Eastment MC, McClelland RS, Mwaringa E, Patta S, et al. The cost of implementing the Systems Analysis and Improvement Approach for a cluster randomized trial integrating HIV testing into family planning services in Mombasa County, Kenya. BMC Health Services Research [Internet]. 2022;22(1):1480.

Reason for exclusion: Ineligible phenomena of interest

Tobin K, Edwards C, Flath N, Lee A, Tormohlen K, Gaydos CA. Acceptability and feasibility of a Peer Mentor program to train young Black men who have sex with men to promote HIV and STI home-testing to their social network members. AIDS Care. 2018;30(7):896–902.

Reason for exclusion: Ineligible phenomena of interest

Tobin R, Crawford G, Hallett J, Maycock B, Lobo R. Utilizing Causal Loop Diagramming to Explore a Research and Evaluation Capacity Building Partnership. Frontiers in public health [Internet]. 2022;10:857918.

Reason for exclusion: Duplicate study

Tobin R, Crawford G, Hallett J, Maycock BR, Lobo R. Critical factors that affect the functioning of a research and evaluation capacity building partnership: A causal loop diagram. PLoS ONE [Electronic Resource] [Internet]. 2022;17(1):e0262125.

Reason for exclusion: Ineligible outcomes

Topp SM, Black J, Morrow M, Chipukuma JM, Van Damme W. The impact of human immunodeficiency virus (HIV) service scale-up on mechanisms of accountability in Zambian primary health centres: a case-based health systems analysis. BMC Health Services Research [Internet]. 2015;15:67.

Reason for exclusion: Ineligible phenomena of interest

Topp SM, Chetty-Makkan CM, Smith HJ, Chimoyi L, Hoffmann CJ, Fielding K, et al. "It's Not Like Taking Chocolates": Factors Influencing the Feasibility and Sustainability of Universal Test and Treat in Correctional Health Systems in Zambia and South Africa. Global health, science and practice [Internet]. 2019;7(2):189–202.

Reason for exclusion: Ineligible phenomena of interest

Topp SM, Mwamba C, Sharma A, Mukamba N, Beres LK, Geng E, et al. Rethinking retention: Mapping interactions between multiple factors that influence long-term engagement in HIV care. PLoS ONE [Internet]. 2018;13(3).

Reason for exclusion: Ineligible phenomena of interest

Tragard A, Shrestha IB. System-wide effects of Global Fund investments in Nepal. Health policy and planning [Internet]. 2010;25 Suppl 1:i58-62.

Reason for exclusion: Ineligible phenomena of interest

Tucker JD, Peng H, Wang K, Chang H, Zhang S-M, Yang L-G, et al. Female sex worker social networks and STI/HIV prevention in South China. PLoS ONE. 2011;6(9):e24816.

Reason for exclusion: Ineligible phenomena of interest

Underhill K, Morrow KM, Colleran CM, Holcomb R, Operario D, Calabrese SK, et al. Access to healthcare, HIV/STI testing, and preferred pre-exposure prophylaxis providers among men who have sex with men and men who engage in street-based sex work in the US. PLoS ONE. 2014;9(11):e112425.

Reason for exclusion: Ineligible phenomena of interest

Van Regenmortel MHV. Development of a Preventive HIV Vaccine Requires Solving Inverse Problems Which Is Unattainable by Rational Vaccine Design. Frontiers in Immunology [Internet]. 2017;8:2009.

Reason for exclusion: Ineligible study design

Veinot TC, Campbell TR, Kruger DJ, Grodzinski A. A question of trust: user-centered design requirements for an informatics intervention to promote the sexual health of African-American youth. Journal of the American Medical Informatics Association. 2013;20(4):758–65.

Reason for exclusion: Ineligible phenomena of interest

Von Rosen FT, Von Rosen AJ, MÃ¼ller-Riemenschneider F, Damberg I, Tinnemann P. STI knowledge in Berlin adolescents. International Journal of Environmental Research and Public Health. 2018;15(1):110.

Reason for exclusion: Ineligible phenomena of interest

Wagner AD, Gimbel S, Asbjornsdottir KH, Cherutich P, Coutinho J, Crocker J, et al. Cascade Analysis: An Adaptable Implementation Strategy Across HIV and Non-HIV Delivery Platforms. Jaids-Journal of Acquired Immune Deficiency Syndromes [Internet]. 2019;82:S322–31.

Reason for exclusion: Ineligible phenomena of interest

Wand H, Falster K, Wilson D, Law M, Maher L. Disproportionate Impact of Combination Antiretroviral Therapy on AIDS Incidence in Australia: Results from a Modified Back-projection Model. AIDS and behavior [Internet]. 2012;16(2):360–7.

Reason for exclusion: Ineligible phenomena of interest

Watts J, O’Byrne P. â◎◎I don’t care if you think I’m gayâ◎¦ that won’t make me either promiscuous or HIV positiveâ◎◎: HIV, stigma, and the paradox of the gay men’s sexual health clinic-An exploratory study. Applied Nursing Research. 2019;47:1–3.

Reason for exclusion: Ineligible phenomena of interest

Wigfall LT, Tomar A, Washington TC. A systems model of integrating HPV self-sampling and evidence-based intervention strategies for increasing follow-up care for abnormal screening results with existing services at community-based HIV/AIDS service organizations. Cancer Epidemiology Biomarkers and Prevention Conference: 13th AACR

Conference on the Science of Cancer Health Disparities in Racial/Ethnic Minorities and the Medically Underserved Virtual [Internet]. 2020;29(12 SUPPL).

Reason for exclusion: Ineligible study design

Wilson PA, Nanin J, Amesty S, Wallace S, Cherenack EM, Fullilove R. Using syndemic theory to understand vulnerability to HIV infection among Black and Latino men in New York City. Journal of Urban Health [Internet]. 2014;91(5):983–98.

Reason for exclusion: Ineligible phenomena of interest

Wilton L, Palmer RT, Maramba DC. HIV and STI Prevention for College Students. In: Understanding HIV and STI Prevention for College Students. 2014. p. 1–13.

Reason for exclusion: Ineligible study design

Windisch R, Waiswa P, Neuhann F, Scheibe F, de Savigny D. Scaling up antiretroviral therapy in Uganda: Using supply chain management to appraise health systems strengthening. Globalization and Health [Internet]. 2011;7 (no pagination).

Reason for exclusion: ineligible publication date

Witzel TC, Rodger AJ, Burns FM, Rhodes T, Weatherburn P. HIV self-testing among men who have sex with men (MSM) in the UK: a qualitative study of barriers and facilitators, intervention preferences and perceived impacts. PLoS ONE. 2016;11(9):e0162713.

Reason for exclusion: Ineligible phenomena of interest

Wohl DA, Khan MR, Tisdale C, Norcott K, Duncan J, Kaplan AM, et al. Locating the places people meet new sexual partners in a southern US city to inform HIV/STI prevention and testing efforts. AIDS and behavior. 2011;15(2):283–91.

Reason for exclusion: Ineligible phenomena of interest

Wong JP-H, Kteily-Hawa R, Chambers LA, Hari S, Vijaya C, Suruthi R, et al. Exploring the use of fact-based and story-based learning materials for HIV/STI prevention and sexual health promotion with South Asian women in Toronto, Canada. Health Education Research. 2019;34(1):27–37.

Reason for exclusion: Ineligible phenomena of interest

Wright PB, Stewart KE, Curran GM, Booth BM. A Qualitative Study of Barriers to the Utilization of HIV Testing Services Among Rural African American Cocaine Users. Journal of Drug Issues [Internet]. 2013;43(3):314–34.

Reason for exclusion: Ineligible phenomena of interest

Wylie JL, Shaw S, DeRubeis E, Jolly A. A network view of the transmission of sexually transmitted infections in Manitoba, Canada. Sexually Transmitted Infections [Internet]. 2010;86 Suppl 3:iii10-16.

Reason for exclusion: ineligible publication date

Xia J, Rutherford S, Ma Y, Wu L, Gao S, Chen T, et al. Obstacles to the coordination of delivering integrated prenatal HIV, syphilis and hepatitis B testing services in Guangdong:

using a needs assessment approach. BMC Health Services Research [Internet]. 2015;15:117.

Reason for exclusion: Ineligible phenomena of interest

Xiong JH, Wang JS, Yang K, Peng SY, Xu QL. Multiagent-Based Simulation of the HIV/AIDS Spatial and Temporal Transmission among Injection Drug Users. In: 18th International Conference on Geoinformatics [Internet]. 2010.

Reason for exclusion: ineligible publication date

Yao K, Wafula W, Bile EC, Cheignsong R, Howard S, Demby A, et al. Ensuring the quality of HIV rapid testing in resource-poor countries using a systematic approach to training. American Journal of Clinical Pathology [Internet]. 2010;134(4):568–72.

Reason for exclusion: Ineligible phenomena of interest

Young I, Flowers P, McDaid LM. Barriers to uptake and use of pre-exposure prophylaxis (PrEP) among communities most affected by HIV in the UK: findings from a qualitative study in Scotland. BMJ Open. 2014;4(11):e005717.

Reason for exclusion: Ineligible phenomena of interest

Zakumumpa H, Rujumba J, Kwiringira J, Katureebe C, Spicer N. Understanding implementation barriers in the national scale-up of differentiated ART delivery in Uganda. BMC Health Services Research [Internet]. 2020;20(1).

Reason for exclusion: Ineligible study design

Zhang JP, Hao WH, Jin Z. The dynamics of sexually transmitted diseases with men who have sex with men. Journal of Mathematical Biology [Internet]. 2022;84(1–2). Available from: <Go to ISI>://WOS:000729775800001

Reason for exclusion: Ineligible phenomena of interest

Zurashvili T, Kasrashvili T, Gogia M. Operational research of barriers and facilitators to harm reduction services for intravenous drug users (Including Female IDUs). HIV Medicine [Internet]. 2019;20(Supplement 9):272.

Reason for exclusion: Ineligible phenomena of interest

de Almeida MC, Araujo FF, Ortega-Jacome GP, Tuboi SH, Fernandes GC. Operational research principles for a routine service process for monitoring the human immunodeficiency virus/ acquired immunodeficiency syndrome treatment cascade: Data from a cohort in Brazil. Revista da Sociedade Brasileira de Medicina Tropical [Internet]. 2017;50(2):229–34.

Reason for exclusion: Ineligible phenomena of interest

de Wit JBF, Adam PCG. A DUAL-SYSTEMS PERSPECTIVE ON SEXUAL HEALTH BEHAVIOR: SELF-CONTROL AS MODERATOR OF ASSOCIATIONS BETWEEN MEN'S IMPLICIT AND EXPLICIT ATTITUDES AND CONDOM USE. International Journal of Behavioral Medicine [Internet]. 2016;23:S5–6.

Reason for exclusion: Ineligible study design
